# EUREC^4^A: A Field Campaign to Elucidate the Couplings Between Clouds, Convection and Circulation

**DOI:** 10.1007/s10712-017-9428-0

**Published:** 2017-09-27

**Authors:** Sandrine Bony, Bjorn Stevens, Felix Ament, Sebastien Bigorre, Patrick Chazette, Susanne Crewell, Julien Delanoë, Kerry Emanuel, David Farrell, Cyrille Flamant, Silke Gross, Lutz Hirsch, Johannes Karstensen, Bernhard Mayer, Louise Nuijens, James H. Ruppert, Irina Sandu, Pier Siebesma, Sabrina Speich, Frédéric Szczap, Julien Totems, Raphaela Vogel, Manfred Wendisch, Martin Wirth

**Affiliations:** 1 LMD/IPSL, CNRS, Sorbonne Université, UPMC, 4 Place Jussieu, 75252 Paris, France; 2 Max Planck Institute for Meteorology, Bundesstr. 53, 20146 Hamburg, Germany; 3 LSCE/IPSL, CNRS-CEA-UVSQ, CEA Saclay, 91191 Gif sur Yvette, France; 4 University of Hamburg, Bundesstrasse 55, 20146 Hamburg, Germany; 5 Woods Hole Oceanographic Institution, 266 Woods Hole Rd, Woods Hole, MA 02543 USA; 6 University of Cologne, Albertus-Magnus-Platz, 50923 Cologne, Germany; 7 LATMOS/IPSL, CNRS-UPMC-UVSQ, 11 Boulevard D’Alembert, 78280 Guyancourt, France; 8 Massachusetts Institute of Technology, 77 Massachussetts Avenue, Cambridge, MA 02139 USA; 9 Caribbean Institute for Meteorology and Hydrology, P.O. Box 130, Bridgetown, Barbados; 10 German Aerospace Center, Múnchener Str. 20, 82234 Oberpfaffenhofen-Wessling, Germany; 11 GEOMAR Helmholtz Centre for Ocean Research, Duesternbrooker Weg 20, 24105 Kiel, Germany; 12 Ludwig-Maximilians University of Munich, Theresienstrasse 37, 80333 Munich, Germany; 13 Delft University of Technology, P.O. Box 5048, 2600 GA Delft, The Netherlands; 14 ECMWF, Shinfield Park, Reading, RG2 9AX UK; 15 LMD/IPSL, Ecole Normale Supérieure, 24 rue Lhomond, 75231 Paris, France; 16 Delft University of Technology and Royal Netherlands Meteorological Institute, De Bilt, Netherlands; 17 Laboratoire de Météorologie Physique, UMR6016, CNRS, Aubière, France; 18 University of Leipzig, Stephanstr. 3, 04103 Leipzig, Germany

**Keywords:** Trade-wind cumulus, Shallow convection, Cloud feedback, Atmospheric circulation, Field campaign

## Abstract

Trade-wind cumuli constitute the cloud type with the highest frequency of occurrence on Earth, and it has been shown that their sensitivity to changing environmental conditions will critically influence the magnitude and pace of future global warming. Research over the last decade has pointed out the importance of the interplay between clouds, convection and circulation in controling this sensitivity. Numerical models represent this interplay in diverse ways, which translates into different responses of trade-cumuli to climate perturbations. Climate models predict that the area covered by shallow cumuli at cloud base is very sensitive to changes in environmental conditions, while process models suggest the opposite. To understand and resolve this contradiction, we propose to organize a field campaign aimed at quantifying the physical properties of trade-cumuli (e.g., cloud fraction and water content) as a function of the large-scale environment. Beyond a better understanding of clouds-circulation coupling processes, the campaign will provide a reference data set that may be used as a benchmark for advancing the modelling and the satellite remote sensing of clouds and circulation. It will also be an opportunity for complementary investigations such as evaluating model convective parameterizations or studying the role of ocean mesoscale eddies in air–sea interactions and convective organization.

## Introduction

Of all the clouds that populate the Earth’s atmosphere, trade-cumuli count among the most fascinating expressions of the interplay between clouds and circulations. These broken shallow clouds form within the lowest kilometres of the atmosphere, influenced at their base by small-scale turbulent motions of the warm, moist surface layer, and at their top by the large-scale sinking motions of the warm and dry overlying free troposphere. If many of these clouds do not rise by more than a few hundred metres above their base, some reach higher levels (e.g Nuijens et al. 2014), detrain and help sustain (evaporatively and radiatively) the trade-wind temperature-inversion layer higher up. Trade-cumuli warm the layer in which they form through condensation, but cool the subcloud layer and the trade inversion through the evaporation of falling raindrops and detrained droplets. In addition, the emission of infrared radiation to space produces an efficient cooling of the lower atmosphere in which clouds form. This radiative cooling contributes to generate shallow mesoscale circulations (Naumann et al. 2017) which, depending on local conditions and remote convective activity, can organize either randomly or into streets, arcs or circles of cloud clusters. In certain conditions, these mesoscale circulations can also trigger remotely the aggregation of deep convection (Muller and Held 2012).

This coupling between shallow clouds and circulation greatly matters for climate sensitivity. Trade-cumuli are so ubiquitous over tropical oceans that their radiative properties substantially influence the Earth’s radiation budget. Their response to global warming is thus critical for global-mean cloud feedbacks, and actually it is their differing response to warming that explains most of the spread of climate sensitivity across climate models (Bony et al. 2004; Bony and Dufresne 2005; Webb et al. 2006; Medeiros et al. 2008; Vial et al. 2013; Boucher et al. 2013; Medeiros et al. 2015). Model diversity in the strength of the vertical mixing of water vapour within the first few kilometres above the ocean surface (in association with both convective and large-scale circulations) is thought to explain half of the variance in climate sensitivity estimates across models (Sherwood et al. 2014): the lower-tropospheric mixing dehydrates the cloud layer near its base at an increasing rate as the climate warms and this rate scales with the mixing strength in the current climate (Sherwood et al. (2014); Gettelman et al. (2012); Tomassini et al. (2015); Brient et al. (2016); Stevens et al. (2016); Vial et al. (2016), Fig. 1). There is increasing evidence that the diversity of the modelled response to warming reflects model diversity in how this coupling between convective mixing, surface turbulent fluxes, and low-cloud radiative effects is represented in regimes of large-scale subsidence, and that it can be partly related to the numerical representation (or parameterization) of convection (Webb et al. 2015; Vial et al. 2016). However, so far it has not been possible to constrain this coupling observationally due to a lack of appropriate measurements.

**Fig. 1:**
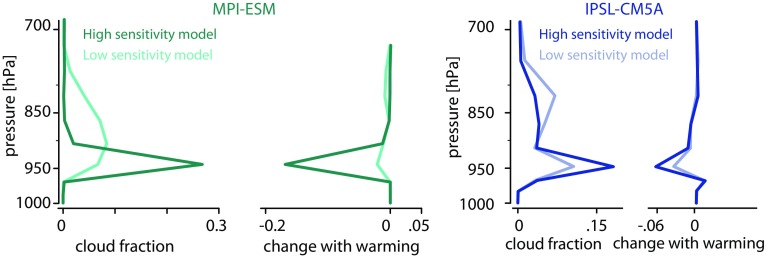
Vertical profiles of the low-cloud fraction, and of its response to global warming, predicted by two general circulation models (MPI and IPSL) in the trade-wind cumulus regime. For each model, results are shown for two versions differing only by their representation of lower-tropospheric mixing (after Stevens et al. 2016; Vial et al. 2016)

On the contrary, in large-eddy simulations (LES) and in observations the cloud-base fraction of trade-wind cumuli appears to be much more resilient to changes in environmental conditions than in climate models, both in the current (Nuijens et al. 2014, 2015a) and projected warmer climate (Rieck et al. 2012; Bretherton 2015). Interpreting these results remains difficult. For the observations, in the past it has not been possible to link cloud amount to the large-scale circulation in which the clouds form. The cloud amount predicted by LES, though often resilient to changes in thermodynamic conditions, is known to be sensitive to various aspects of the simulation such as resolution, microphysics, numerics or domain size (Vial et al. 2017, and references therein). Theoretically, the apparent resilience of cloud-base cloud fraction has been interpreted as the consequence of a “cumulus-valve mechanism” whereby clouds act as a valve which helps maintain the top of the subcloud layer close to the lifting condensation level and thus regulate the area covered by cumulus updrafts at cloud base (Albrecht et al. 1979; Neggers et al. 2006; Stevens 2006; Nuijens et al. 2015a). However, this idea has not been tested observationally. Moreover, recent studies running large-eddy simulations over large domains question this idea of cloud-base resilience, as they show that changes in the mesoscale organization of shallow cumuli can significantly influence the cloud fraction (Seifert and Heus 2013; Vogel et al. 2016; Vial et al. 2017). It is thus paramount to assess the ability of LES to predict the cloud cover and its dependence on the organization of convection and on environmental conditions.

The discussion above illustrates how the science has matured to the point where it is now possible to identify a few key hypotheses or questions that, if tested or answered, would enable a step improvement in understanding of the interplay between clouds, convection and circulation, and their role in climate change: How strong is the convective mixing in regimes of shallow cumulus and how much does it couple to surface turbulent fluxes, radiative effects and water vapour? Is the cloud-base fraction of trade-wind cumuli insensitive to variations in convective mixing and large-scale circulations? Does the cumulus mass flux act as a valve to restrict the turbulent boundary layer from growing appreciably beyond its lifting condensation level? Do the statistics of shallow convection depend on the form of spatial organization?

Improved observations are also necessary to help advance space-based remote sensing. The trade-wind regions are often characterized by a strongly layered vertical structure, and by warm, small, and thin broken clouds. Current satellite observations are inadequate to detect sharp vertical gradients of water vapour (Chazette et al. 2014; Asrar et al. 2015, Stevens et al. this volume), and the detection of shallow clouds from space remains difficult. Biases in cloud detection leads to significant discrepancies among the various satellite estimates of the trade-wind cloud fraction (Stubenrauch et al. 2013) and are detrimental to the quality of other satellite retrievals such as those of the cloud water path (Horváth and Gentemann 2007), precipitation and cloud microphysical properties. In these conditions, in situ observations are not only critical to investigate the physics of trade-wind clouds, but also to test—and eventually improve—the instruments and algorithms of remote sensing that are used to observe the Earth’s atmosphere and surface from space.

Past field campaigns in regions of shallow cumulus such as the *Atlantic Expedition* in September to October 1965 (Augstein et al. 1973), the *Atlantic Tradewind EXperiment* in February 1969 (ATEX, Augstein et al. 1974), the *Barbados Oceanographic and Meteorological Experiment* from May to July 1969 (BOMEX, Holland 1970) or the *Puerto-Rico Experiment* in December 1972 (LeMone and Pennell 1976), did focus on the environment of clouds, on vertical transports of water, heat and momentum in the trade-wind boundary layer, and also included attempts to measure the large-scale vertical motion in the atmosphere. However, the microphysical and macrophysical properties of the shallow cumuli were not characterized. Because these campaigns took place at the dawn of the satellite era, no observations from space could help fill the gap. In June 1992, the *Atlantic Stratocumulus Transition Experiment* (ASTEX, Albrecht et al. 1995) was conducted off North Africa, in the area of Azores and Madeira Islands, to address issues related to the stratocumulus to trade-cumulus transition and cloud-mode selection. Satellites and upper-level aircraft provided a description of large-scale cloud features, and instrumented aircraft flying in the boundary layer and surface-based remote sensing systems described the mean, turbulence, and mesoscale variability in microphysical properties of boundary-layer clouds. Attempts were also made to infer large-scale divergence in the boundary layer using lagrangian balloons and an array of three rawinsonde stations (Ciesielski et al. 1999). In 2005, the *Rain in shallow cumulus over the ocean* (RICO, Rauber et al. 2007) campaign which took place off the Caribbean islands of Antigua and Barbuda did focus on cloud microphysical properties and pointed out the importance and diversity of mesoscale organizations, but the large-scale dynamical environment and the interplay between cloud macrophysical properties (e.g., the low-level cloud fraction) and their environment were not characterized.

The establishment of the Barbados Cloud Observatory (BCO) in 2010, and the two *Next-Generation Aircraft Remote Sensing for Validation Studies* airborne field campaigns (NARVAL and NARVAL2) held in December 2013 and August 2016 have since created an observational foothold to better understand the coupling between clouds and their environment (Stevens et al. 2016). The BCO provides long-term context for intensive observations, and when combined with the NARVAL measurements, helps advance and test new approaches for bridging the gap between measurements of cloud macro-structure and the large-scale environment.

A new field campaign, EUREC^4^A (*Elucidating the role of clouds-circulation coupling in climate*), has been designed to take advantage of and extend these advances. Anchored at the BCO, it will measure clouds in the winter trades of the North Atlantic, windward of Barbados, in early 2020. EUREC^4^A will have two primary objectives:

- To quantify macrophysical properties of trade-wind cumuli as a function of the large-scale environment, and
- To provide a reference data set that may be used as a benchmark for the modelling and the satellite observation of shallow clouds and circulation.

To address these objectives EUREC^4^A will provide, for the first time, simultaneous measurement of cloud macrophysical properties (cloud fraction, vertical extent and cloud-size distributions), cloud radiative properties (large-scale albedo, broadband solar and terrestrial net fluxes and derived quantities such as radiative divergence and heating/cooling rates), convective activity (cloud-base mass flux, mesoscale organization), and the large-scale environment in which clouds and convection are embedded (large-scale vertical motion, thermodynamic stratification, surface properties, turbulent and radiative sources or sinks of energy).

In Sect. 2, we present an overview of the experimental strategy for the EUREC^4^A field campaign. In Sect. 3, we discuss the premises which are at the basis of this strategy, namely the possibility to measure cloud profiles (especially cloud amount at cloud base), convective mass flux and large-scale vertical velocity, as only this can connect the macrophysical properties of clouds to the environment. Then, we discuss how the results from the campaign could be used to build a reference data set for evaluating process and climate models, and for assessing retrievals from space-borne observations (Sect. 4). Beyond the study of clouds-circulation interactions, EUREC^4^A will be an opportunity for complementary scientific investigations. We describe some of these possibilities as of EUREC^4^A^++^ in Sect. 5. A brief conclusion is presented in Sect. 6.

## Overview of the EUREC^4^A Experimental Strategy

The core objective of the EUREC^4^A field campaign is to elucidate how the macrophysical properties of trade-cumuli depend on the dynamic and thermodynamic properties of the environment in which the clouds form. More specifically, EUREC^4^A aims to answer the following questions:

- What controls the convective mass flux, mesoscale organization, and depth of shallow clouds?
- How does the cumulus cloud amount in the trade-wind boundary layer vary with turbulence, convective mixing and large-scale circulations, and what impact does this variation have on the atmospheric radiation field?

**Fig. 2:**
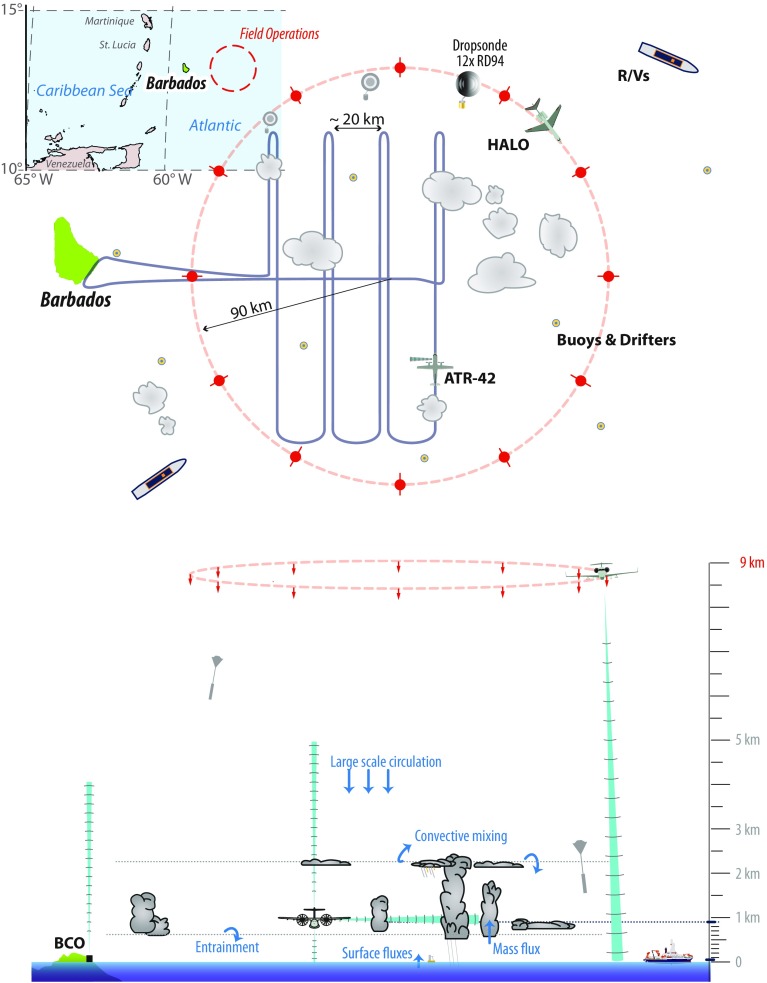
Envisioned flight strategy for the EUREC^4^A core measurements

The EUREC^4^A field campaign will take place in the lower Atlantic trades, over the ocean east of Barbados ($13\,^\circ \hbox {N}, 59\,^\circ \hbox {W}$) from 20 January to 20 February 2020. Several reasons motivate the choice of this specific location. First, shallow cumuli are prominent in this area, especially during winter (Norris 1998; Nuijens et al. 2014). Second, the cloudiness in the vicinity of Barbados is representative of clouds across the whole trade-wind regions of the tropical ocean, both in models and in observations (Medeiros and Nuijens 2016). Finally, it anchors the measurements to the extensively instrumented Barbados Cloud Observatory which has been monitoring clouds continuously since 2010, and allows it to benefit from the legacy of the NARVAL series of flight campaigns organized in the area in the last few years (Stevens et al. 2016).

### Aircraft Measurements

The primary motivation for EUREC^4^A is the need to characterize simultaneously the trade-cumulus field and the dynamic and thermodynamic environment in which it forms. For this purpose, the core of the EUREC^4^A field campaign will be the deployment of two research aircraft (Fig. 2): The French ATR-42 operated by the Service des Avions Français Instrumentés pour la Recherche en Environnement (SAFIRE), which will fly in the lower-troposphere with a payload of up to two tons and will be equipped with both remote sensing instrumentation and a suite of in situ sensors (Table 1), and the German HALO (High Altitude and Long Range Research Aircraft) operated by the Deutsches Zentrum für Luft- and Raumfahrt (DLR), which has a payload of up to three tons, a range of up to 8000 km, and a ceiling of up to 15 km, an advanced instrumentation (Table 2) and the ability to launch dropsondes (see for instance Wendisch et al. 2016). In addition to these aircraft, we will use the Barbados Cloud Observatory (BCO), buoys, drifters plus several research vessels deployed in the area and equipped with radiosondes and additional remote sensing instruments to complement surface, atmospheric and ocean measurements (Sect. 2.2).

HALO will fly large circle patterns (45–50 min, corresponding to a circumference of about 500 km) at 9 km altitude (flight level 300, FL300), and will densely distribute dropsondes around the circles. The dropsondes will characterize the vertical thermodynamic structure of the trade-wind atmosphere and will make it possible to infer the vertical profile of large-scale divergence over the area (Sect. 3.1). The advanced remote sensing instrumentation on HALO will characterize the cloud field and its environment (water vapour, hydrometeors, cloud particle phase, cloud vertical structure, cloud albedo, etc).

**Table 1:** Synopsis of ATR-42 instrumentation

Instrument	Brief description
Thermodynamics and turbulence	In situ water vapour, temperature, pressure and 3D wind; momentum and heat fluxes
Cloud particles	In situ liquid and total water contents; droplet size distribution (0.5–6000 μm); 2D particle imaging (25–6000 μm)
BASTA cloud radar	Bistatic 95 GHz Doppler cloud radar to be deployed in sidewards looking mode (Delanoë et al. 2016)
ALiAS Lidar	Lightweight backscatter lidar (355 nm) to be deployed in sidewards looking mode (Chazette 2016)
RASTA cloud radar	Upward- and downward-looking 95 GHz Doppler cloud radar with six antenna configuration for wind-vector retrievals (Delanoë et al. 2013)
LNG lidar	Three-wavelength (1064, 532 and 355 nm) high-spectral-resolution polarized backscatter lidar (upwards, downwards or 35° pointing) (Bruneau et al. 2015)
CLIMAT-AV	Three-channel downward-staring measurements of infrared irradiance at 8.7, 10.8, and 12.0 μm (Brogniez et al. 2003)
Pyrgeometer	Hemispheric broadband upwelling and downwelling thermal infrared radiative fluxes (Kipp and Zonen CGR4)
Pyranometer	Hemispheric broadband upwelling and downwelling solar radiative fluxes (Kipp and Zonen CMP22)

Simultaneously, the ATR-42 will characterize the shallow cumulus field and boundary-layer properties within the area through a series of low-level legs, flown primarily near the cloud-base level ($\sim 1\,\hbox {km}$), with additional legs near the trade inversion level ($\sim 2\,\hbox {km}$), and (by flying at the lowest safe flight level) near the sea surface. Sideways-looking lidar and radar instruments will measure the cloud fraction at the flight level (Sect. 3.2). Upward-pointing high-spectral-resolution (HSR) backscatter lidar plus a vertically pointing Doppler radar will be used to assess the boundary-layer depth and measure the vertical velocity in the aerosol-laden lower troposphere above the aircraft. Other instruments on board the aircraft will characterize cloud microphysical properties, the tri-dimensional wind field along the trajectory of the aircraft, and turbulence statistics (Table 1, Appendix 1).

**Table 2:** Synopsis of HALO instrumentation

Instrument	Brief description
BAHAMAS	In situ water vapour, temperature, gust probe winds and aircraft state vector. Up- and downward shortwave and longwave broadband irradiances (in development)
HAMP cloud radar	Downward-staring polarized Doppler 36 GHz cloud radar (Mech et al. 2014)
HAMP radiometer	Downward-staring microwave radiometers with 26 channels between 22 and 183 GHz (Mech et al. 2014)
WALES	Downward-staring water vapour DIAL and backscatter HSRL lidar (Wirth et al. 2009)
SMART	Up- and downward-looking spectral (300–2200 nm) radiance and irradiance measurements (Wendisch et al. 2001; Ehrlich et al. 2008)
SpecMACS	Downward-looking hyper-spectral (400–2500 nm) line imager (Ewald et al. 2016)
Thermal imager	Downward-looking (10.8 and 12 μm) two channel line imager (in development)
Dropsondes	AVAPs system with four-channel receiver supporting Vaisala RD94 Sondes (ten channel receiver in development)

The instrumentation on board both aircraft will provide a detailed characterization of the vertical distribution of water vapour, clouds and aerosol particles, and of vertical velocities within clouds (Appendix 1). Measurements of radiative fluxes at different altitudes, as well as radiative transfer calculations using observed atmospheric and cloud properties, will help infer vertical profiles of radiative solar heating and terrestrial cooling rates above, within and below the observed clouds.

The characterization of surface (Sect. 2.2) and subcloud layer properties, and of the difference between the subcloud layer and the air just above it, combined with estimates of surface turbulent fluxes, radiative cooling and large-scale mass divergence, should enable closure of the mass and moist static energy budgets of the subcloud layer. The analysis of the mass budget will make it possible to estimate the cumulus mass flux at cloud base (Sect. 3.3). The moist static energy and water budgets will be used to verify the consistency among the different measurements and provide insight into the factors influencing shallow convective development. The moist static energy budget should also make it possible to test the boundary-layer quasi-equilibrium hypothesis (Raymond 1995), which holds that the cumulus enthalpy flux out of the subcloud layer balances surface fluxes and large-scale ascent at the top of the subcloud layer.

To maximize the chance of sampling a large diversity of environmental conditions and mesoscale organizations, the campaign will consist of about 90 h of research flights (for each aircraft) over four weeks for operations out of Grantley Adams International Airport on Barbados. Ten HALO research flights from Barbados are envisioned, each with a duration of 9 h bracketing two 4-h flights of the ATR-42 (with a refuelling in between). The zone of operations will not change much from day to day, but owing to daily meteorological variability one may hope to sample different types of cloud conditions and organizations. On any given research flight the same cloud and environmental conditions will be observed for many hours by both aircraft, making it possible to characterize these conditions in a statistically consistent and representative way.

### Surface and Ship-Based Observations

In addition to the aircraft missions that will characterize clouds and their surrounding environment up to scales of O(100 km), surface and ship-based observations will be distributed over the area of and around flight operations augmenting measurements from the BCO so as to better characterize the surface and atmospheric environment of clouds on a scale of O(1000 km) and over a longer, uninterrupted time period, as well as strengthen the fidelity of the large-scale analyses.

**Fig. 3:**
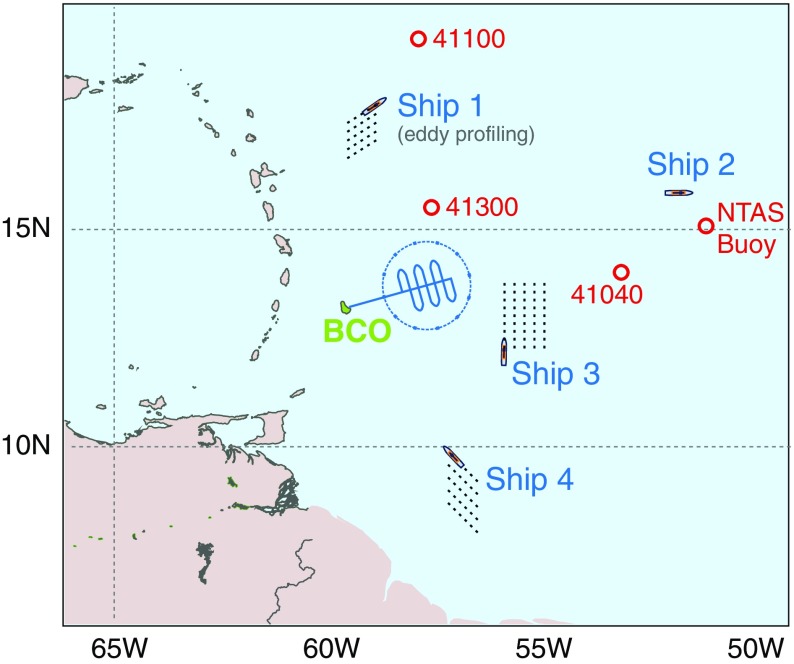
Large-scale sounding array envisioned for EUREC^4^A, comprised by the Barbados Cloud Observatory (BCO) and approximately four research vessels. Buoy stations are indicated by open circles. The ships will serve as advanced surface remote sensing platforms, atmosphere and ocean sounding stations, bases for fleets of autonomous vehicles, and means of laying down an array of drifters/floats or buoys

The large-scale array of observations will be comprised of three to five stations (Fig. 3): the Barbados Cloud Observatory (BCO, Stevens et al. 2016) and a network of research vessels (RVs). Applications for ship measurement time from Germany (*Meteor* and *Maria S. Merian*), France (*Atalante*), The Netherlands (*Pelagia*), the USA and Spain are pending. The research vessels will serve as advanced surface remote sensing platforms, atmosphere and ocean sounding stations, bases for fleets of autonomous vehicles, and means of laying down an array of drifters/floats or buoys.

Radiosondes will be launched from each station to collect simultaneous measurements of profiles of air humidity, temperature, pressure and horizontal winds (derived from GPS measurements) from the surface through the lower stratosphere. The sounding data will be assimilated by weather centres, which will improve the quality of meteorological analyses for the period of the campaign and will help diagnose large-scale divergence and vertical motion over a range of scales larger than the zone of aircraft operations.

Measurements from research vessels are intended to be operated over a period of 3 to 5 weeks overlapping with the planned airborne observations. This will establish context for the aircraft missions relative to the seasonal march of the ITCZ and the evolving strength of overturning within the Hadley cell. Soundings will be launched with a minimum frequency of 6 per day to adequately sample the diurnal and semi-diurnal cycles, along with other subdaily variability. It will also make it possible to get thermodynamic data and large-scale divergence estimates up to the top of the atmosphere instead of up to 9 km (the flight level of HALO during EUREC^4^A).

Besides radiosondes, in situ and remote sensing instrumentation will be installed at BCO and on-board the ships. Instruments such as lidar, radar, radiometers or ceilometers will provide additional observations of clouds, aerosols, surface turbulence and air–sea fluxes of heat and moisture, and surface and boundary-layer properties. A scanning, S-band, radar operated by the Barbados Meteorological Service can be used for research purposes in the absence of severe weather. It will help characterize the mesoscale organization of convection, and the vertical structure of the shallow cloud cover (Nuijens et al. 2009; Oue et al. 2016). The deployment of a second C-Band radar, the POLDIRAD of the Institut für Physik der Atmosphäre at the Deutschen Zentrum für Luft- und Raumfahrt, is also being considered. Shipboard deployment of drones and a HeliKite, capable of suspending an instrument of up to 100 kg at different heights in the lower 3 km of the atmosphere, can be used to characterize aerosol and cloud microphysical properties. Laser-based spectrometers could measure the isotopic composition of water and provide an additional characterization of the balance between convective drying and turbulent moistening in the boundary layer, and simple instruments such as ceilometers will aid the characterization of the vertical distribution of clouds in the observational domain.

The ships will also provide an opportunity to characterize the state of the upper ocean and more specifically the mesoscale ocean eddies which are particularly frequent east of Barbados (Sect. 5.6). Beyond their importance for the ocean transport, mesoscale ocean eddies are increasingly recognized as influencing air–sea fluxes and clouds (Chelton et al. 2004; Ferreira and Frankignoul 2008; Frenger et al. 2013; Byrne et al. 2015). This raises the question as to whether they might play a role in the organization of shallow cumuli. Oceanographic measurements of the vertical profiles of temperature, salinity, pressure, oxygen and other biogeochemical properties of the upper ocean through in situ sensors or profiling instruments, combined with the deployment of Argo profiling floats and autonomous observing platforms such as gliders or wave-gliders, would provide an unprecedented characterization of tropical mesoscale ocean eddies under a well-observed atmosphere and would thereby foster studies of their impact on air–sea interaction (Sect. 5.6).

### Satellite Observations

To complement the airborne measurements, we will coordinate field operations with overpasses of several satellites: the Advanced Spaceborne Thermal Emission and Reflection Radiometer (ASTER), an imaging instrument with 15 m spatial resolution on-board Terra, plus a number of satellites from flagship space missions that we expect to be in orbit by the time of the campaign: *ADM-Aeolus* (whose launch is planned by the beginning of 2018) will provide the first space-borne vertically resolved radial (mostly zonal) wind measurements; *EarthCare* (Illingworth et al. 2015, whose launch is scheduled in 2019), includes a Doppler cloud radar and a HSR lidar which will provide a thorough characterization of clouds and aerosols from space-based products comparable to those issued from the radar and the HSR lidar on-board the ATR-42 at the same frequency and wavelength; and *Megha-Tropiques* (Roca et al. 2015, launched in 2011, the mission has been extended until 2021) measures radiative fluxes at the top of the atmosphere, the vertical distribution of relative humidity through the troposphere, and precipitation as part of the GPM (Global Precipitation Measurement) mission.

**Fig. 4:**
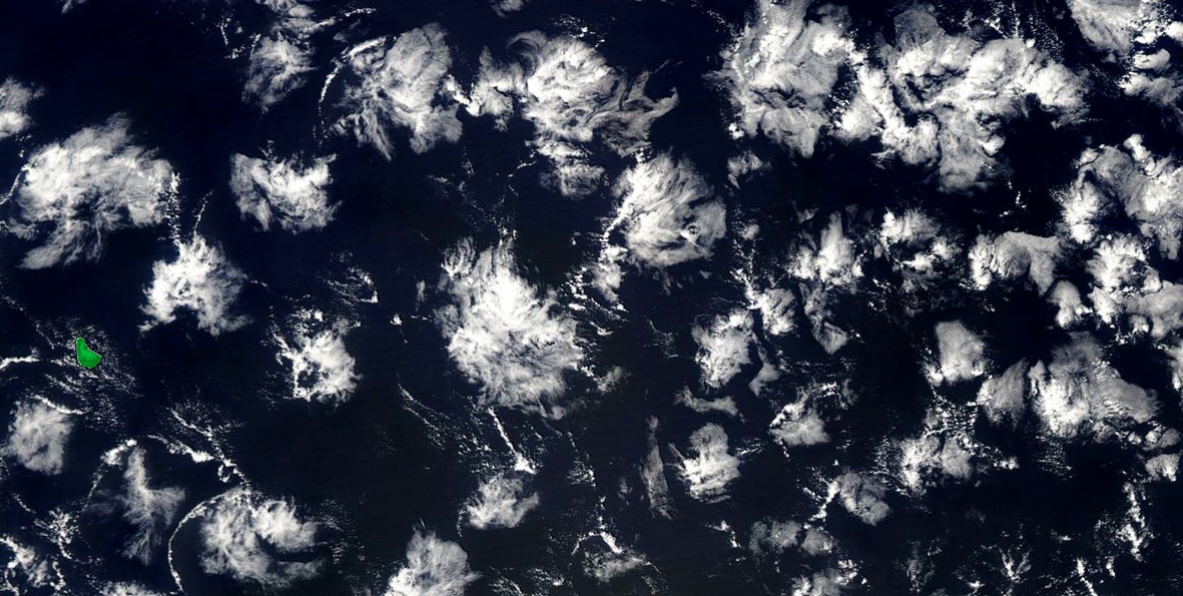
Shallow cloud organization observed from MODIS on 9 February 2017 (Barbados, which is about 20 km wide, is highlighted in green). The top of cloud clusters does not exceed 3–4 km

Existing satellite imagery suggests that shallow cumuli exhibit a large range of mesoscale organizations from seemingly randomly distributed cloud clusters to wind-parallel street lines or arcs (Rauber et al. 2007). An example of one form of organization, mesoscale cloud flowers, observed over the proposed EUREC^4^A study area on 9 February 2017 is shown in Fig. 4. Space observations of the atmosphere at high spatial resolution such as derived from ASTER or other instruments such as the Geostationary Operational Environmental Satellite (GOES), Multi-angle Imaging SpectroRadiometer (MISR) or Moderate Resolution Imaging Spectroradiometer (MODIS) imagers, potentially complemented by radar observations from the surface network (Sect. 2.2), will characterize the spatial organization of clouds within the area sampled by the aircraft missions. EUREC^4^A will be the first field study to investigate whether this organization matters for the statistical properties of the shallow cumulus field.

## The Premises

The experimental strategy of the EUREC^4^A campaign rests on three main premises:

- The large-scale vertical motion on scales O(100 km) can be measured using dropsondes,
- The distribution of clouds in the trade-wind boundary layer can be inferred from lidar-radar measurements, especially the cloud fraction near cloud base,
- The convective mass flux at cloud base can be inferred from the subcloud layer mass budget.

In this section, we present arguments and results from ongoing analyses that show that the first of these premises appears sound, and we discuss how the other two are currently being tested, and how the experimental strategy might be adapted based on the outcome of these tests.

### Using Dropsondes to Measure the Large-Scale Vertical Motion

A main component of the large-scale mass, heat and moisture budgets is the large-scale vertical velocity $\omega$ (Yanai et al. 1973). From the equation of mass continuity, $\omega$ can be derived from the divergence, *D* of the horizontal wind ${\mathbf {V}}_{\mathrm {H}}$ as $\omega (P) = - \int _0^P D(p) \, {\mathrm {d}}p$ where $D = \nabla \cdot {\mathbf {V}}_{\mathrm {H}}$ and *P* is the atmospheric pressure. *D* and $\omega$ are known to strongly influence the properties of the trade-cumulus boundary layer and low-level cloudiness (e.g., Albrecht et al. 1979). Our ability to measure these two quantities during EUREC^4^A will thus critically determine the success of the campaign.

Measurements of the large-scale vertical motion on the time and space scale of individual airborne observations have long been recognized as being essential to understand how cloudiness develops and to calculate the heat and moisture budgets of the lower troposphere, but so far generally thought to be impossible. During ATEX, BOMEX, ACE-I and DYCOMS-II campaigns, attempts were made to estimate the large-scale divergence from rawinsonde sounding networks and/or aircraft data at a particular level using the “line integral” method (Holland and Rasmusson 1973; Nitta and Esbensen 1974b; Lenschow et al. 1999, 2007). This method infers *D* from horizontal wind measurements using:

1 $$\begin{aligned} D = \frac{1}{A}\,\oint V_n\,{\mathrm {d}}\ell , \end{aligned}$$

where $V_n$ is the component of the horizontal wind normal to the perimeter of measurements, and *A* is the area of the region enclosed by it (vorticity can be obtained similarly from the tangent component of the horizontal wind). When applied to aircraft measurements, this method requires a stationary wind field but makes no other assumption about the structure of the wind field.

An alternative method, referred to as the “regression method”, has been proposed by Lenschow et al. (2007) and successfully applied to DYCOMS-II data. It assumes a particular model for the wind field, but can be more easily adopted to a wider range of sampling geometries. Lenschow et al. (2007) assumed that wind variations in longitude, latitude and time are linear for each vertical level, such that:

2 $$\begin{aligned} {\mathbf {V}}_{\mathrm {H}} = {\mathbf {V_o}} + \frac{\partial {\mathbf {V}}_{\mathrm {H}}}{\partial x}\,{\Delta }x + \frac{\partial {\mathbf {V}}_{\mathrm {H}}}{\partial y}\,{\Delta }y + \frac{\partial {\mathbf {V}}_{\mathrm {H}}}{\partial t}\,{\Delta }t , \end{aligned}$$

where ${\mathbf {V_o}}$ is the mean wind velocity over the area, ${\Delta }x$ and ${\Delta }y$ are the eastward and northward displacements from a chosen centre point. ${\Delta }t$ is the change in time relative to a reference, for instance the mid-point time of the sampling. An approximate solution of this overdetermined system can be found by computing the coefficients of a least squares fit to the wind field defined as (2). By measuring ${\mathbf {V}}_{\mathrm {H}}$ and solving (2) for its gradients, *D* can then be computed as: $D = \frac{\partial u}{\partial x} + \frac{\partial v}{\partial y}$.

So far, these methodologies have been applied to wind measurements from rawinsondes or flight-level estimates of winds from an aircraft gust probe. Wind measurements from GPS dropsondes (Wang et al. 2015) now offer the opportunity to measure the vertical profiles of *D* and $\omega$ during airborne field campaigns. However, this methodology needs to be evaluated. In particular, it has to be checked whether the divergence measured in this way would actually represent the large-scale circulation or would instead be noisy and dominated by short-term features uncharacteristic of the large-scale environment.

**Fig. 5:**
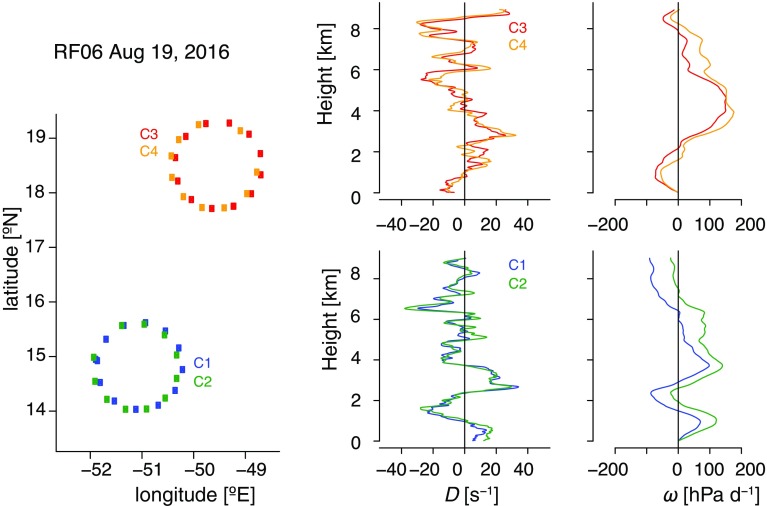
(left) Research flights performed during NARVAL2 on 19 August and the vertical profiles of large-scale mass divergence *D* and large-scale vertical velocity $\omega$ derived from the dropsondes measurements for each circle

To answer this question, this methodology was tested during the NARVAL2 campaign, which consisted of ten research flights of HALO, and took place in the EUREC^4^A target areas, upwind of Barbados, during August 2016. Two of the HALO flights during NARVAL2 were specifically designed as a pilot study for the proposed EUREC^4^A divergence measurements. During the two research flights RF03 and RF06 (carried out on 12 and 19 August 2016), HALO flew horizontal circles of 45–48 min (160 km diameter) at an altitude of 9 km. Twelve dropsondes (Vaisala RD94) were released intensively along each circle, measuring the vertical profiles of pressure, temperature and humidity with an accuracy of 0.4 hPa, $0.2 \,^\circ \hbox {C}$ and 2%, respectively. Equipped with a GPS receiver, the dropsondes also measured the horizontal wind speed with an accuracy of $0.1 \, \hbox {m.s}^{-1}$.

To test the method, pairs of circles were flown in the same air mass (one clockwise, one counterclockwise, with the centre of the second circle slightly displaced following the mean wind relative to the first one). The idea was that if the wind field was sufficiently stationary, and the measurements by the sondes were physical, one would expect similar answers to arise between a pair of circles flown in the same airmass. Satellite imagery targeted the flights to regions of suppressed convection, with a relatively more active shallow cloud layer during the second pair of circles, with cloud tops reaching sometimes 2–3 km, than during the first pair, with cloud tops rarely exceeding 1.5 km (Bony and Stevens Measuring large-scale vertical motions with dropsondes, manuscript in preparation). As shown in Fig. 5, the vertical profiles of *D* and $\omega$ derived for each circle of a given pair exhibit a consistent and reproductible vertical structure over most of the troposphere. Differences between circles of a given pair are much smaller than differences from one pair to the next, where different pairs of circles were spatially dislocated. The vertical structure of *D* and $\omega$ measured by dropsondes in the lower troposphere (below 4 km), such as the maximum subsidence near the top of the mixed layer, is qualitatively consistent with that measured by rawinsondes or aircraft measurements during previous field campaigns in the trades (Holland and Rasmusson 1973; Nitta and Esbensen 1974b). It is also in good agreement with the vertical structure of *D* and $\omega$ derived over the area from ECMWF operational forecasts during periods where the horizontal wind of the forecasts is in good agreement with the dropsondes, and with storm-resolving (1 km grid) simulations initialized by ECMWF analyses (not shown). It shows therefore that dropsondes can actually be used to measure the vertical profiles of *D* and $\omega$ on scales of O(100 km) and to discriminate the spatial heterogeneity of the environment.

Three further issues are currently being explored, also in combination with high-resolution simulations which will be used to emulate different sounding strategies: (1) The minimum number of sondes to be dropped along each circle to reach equivalent results, (2) the spatial scale over which the large-scale dynamics best correlates with the macrophysical cloud properties, and (3) the influence of vertical shear of the horizontal wind, which is much more pronounced during the winter season. Depending on the result of these investigations, the number of sondes to be dropped, as well as the size of the circular flights to be flown during EUREC^4^A will be optimized.

### Estimating the Distribution of Clouds in the Trade-Wind Boundary Layer

An additional important and novel element of the EUREC^4^A strategy will be to measure the cloud fraction within the trade-wind boundary layer, especially around two critical levels: just above cloud base (around 1 km) and around the trade-inversion level (around 2 km).

**Fig. 6:**
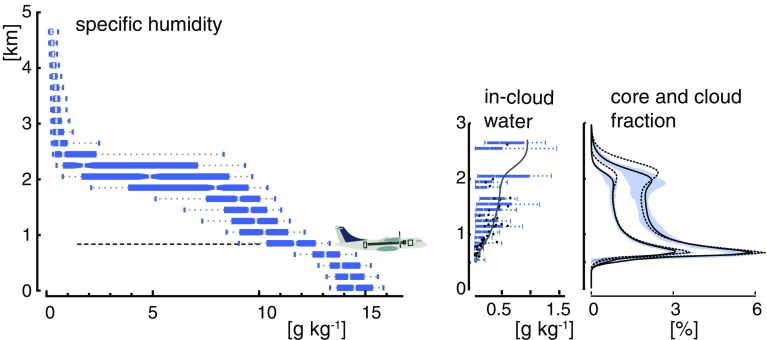
Vertical profiles of water vapour mixing ratio (left) from the NARVAL 1 flights, (middle) condensed water ($q_l$) and (right) cloud fraction from RICO. The NARVAL 1 water vapour is derived from all sondes for which surface air temperatures exceed $25^{\circ } \hbox {C}$, as measured east of Barbados in December 2013. The distribution is described by the box plots showing range (5–95%), interquartile and median. Cloud condensate profiles are for similar conditions but during RICO and adapted from vanZanten et al. (2011). Flight date is characterized by box plots (interquartile and 5–95%) and dots (flight averaged). The line is the ensemble mean of 12 large-eddy simulations. Cloud and cloud-core fraction profiles are derived from LES simulations (adapted from vanZanten et al. 2011). Ensemble (interquartile) spread among LES simulations is given by the shading, and the mean profiles from non-precipitating simulations are shown by the thin dashed line. Approximate flight level for the cloud-base legs of the ATR-42 is also indicated

Measurements of cloud fraction at cloud base are important for understanding what processes control its variations in the current climate and to test some of the processes involved in the climate change cloud feedbacks of climate models. However, upper-level clouds masking the field of view, they are difficult to make with downward-looking instruments. During EUREC^4^A, we propose to use the ALiAS backscatter lidar (Chazette et al. 2007; Chazette 2016) and the Bistatic Radar System for Atmospheric Studies (BASTA) radar (Delanoë et al. 2016) on-board the ATR-42 flying just above cloud base to acquire dedicated horizontally pointing observations from the aircraft windows. At this level, LES (e.g., vanZanten et al. 2011; Vogel et al. 2016) suggest that the relative dryness of the atmosphere combined with the relatively low cloud water content (Fig. 6) should maximize the range of the lidar measurements, thereby providing useful backscatter signal over a distance of about 10 km, thus greatly enhancing the sampling volume. Beyond the mean cloud fraction, the lidar-radar measurements will help determine the spectrum of cloud sizes at cloud base, which is thought to be a crucial information for understanding the coupling between the subcloud layer and the cloud layer (Neggers 2015).

**Fig. 7:**
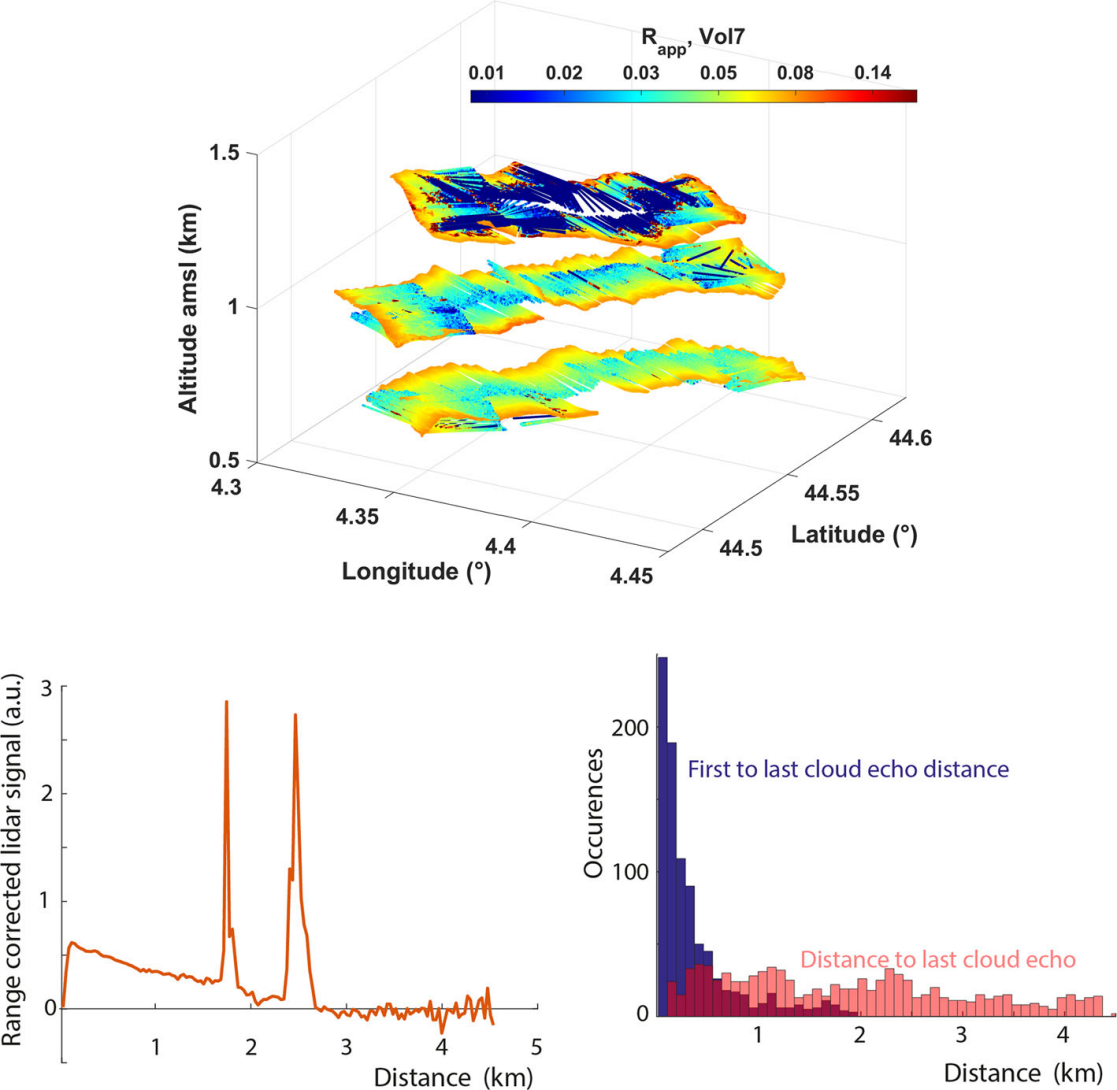
(Top) Lidar backscatter ratio measured on 2 June 2017 (RF07), in the South of France from an ultra-light aircraft carrying a 355-nm horizontally pointing lidar: two rectangular legs were flown within the subcloud layer and one above the base of shallow cumuli. (Bottom left): Example of an individual lidar signal (corrected from aerosol attenuation) detecting two clouds in a row. (Bottom right): Histogram of the distance between the first and last cloud detections along each individual lidar beam and of the distance between the ULA and the last detected cloud. Note that the ALiAS lidar that will be on-board the ATR-42 will be five times more powerful than the lidar used on-board the ULA

To test the approach, measurements from a field campaign which took place on 1–6 June 2017 in Ardèche (South of France, 44.4° N, 4° E) and during which a lidar was mounted horizontally on an Ultra-Light Aircraft (ULA) are being analysed. The lidar, an eye-safe 355-nm backscatter lidar similar to the ALiAS lidar (Chazette 2016) to be used during EUREC^4^Abut five times less powerful (6 vs. 30 mJ), was pointing horizontally towards the port side of the ULA. The ULA flew a series of horizontal legs of rectangular shape within the subcloud layer and above cloud base within a field of shallow cumuli (Fig. 7). Preliminary analysis of the data suggests that above cloud base (at an altitude of about 1.2 km), the lidar signal does not saturate or vanish as soon as it encounters the first cloud edge along the line of sight, but often penetrates clouds over several hundred metres (100–200 m on average for opaque clouds) and can even go through several consecutive clouds when clouds are optically thin. Overall, this low-power lidar makes it possible to detect the presence of shallow cumuli over a distance of up to 4.5 km. Considering that the ALiAS lidar on board the ATR-42 will be five times more powerful than this one, one may hope to detect clouds over a distance of about $4.5 \sqrt{5} = 10 \, \hbox {km}$ during EUREC^4^A, thus making it possible to map the cloud field in between ATR-42 legs spaced by about 20 km. The use of two gated detectors for different ranges on the lidar (one measuring near-field signals and the other far-field signals) is being considered to enhance the cloud detection range. The combination of lidar and radar measurements should further improve the restitution of the cloud mask over this distance.

The feasibility of the approach will be further tested using LES and by applying to LES outputs the McRALI (Monte Carlo Radar and LIdar) simulator of the lidar and radar instruments on board the ATR-42. McRALI is a forward Monte Carlo model (Cornet et al. 2010) enhanced to take into account light polarization, multiple scattering, high spectral resolution, Doppler effects and the three-dimensional structure of the cloudy atmosphere (Szczap et al. 2013, Alkasem et al. 2017). By diagnosing the cloud fraction that the lidar and radar would measure if they were probing an atmosphere similar to that simulated by the large-eddy model, we will assess how well the above approach can work, and/or whether the experimental strategy will have to be revised to get more accurate measurements of the cloud-base cloud fraction.

Besides the cloud base information, it will be important to determine the vertical profile of cloud fraction, especially at the top of the cloud layer. Indeed the cloud fraction near the inversion level appears to be more variable than that at cloud base (Nuijens et al. 2014), varies strongly with the intensity of the convective mass flux (e.g., Brient et al. 2016; Vial et al. 2016) and strongly influences the variations of the total cloud cover (Rodts et al. 2003). The cloud fraction near the inversion level will be estimated through different methods. First, the ATR-42 could fly around this level and measure the cloud mask through horizontal lidar/radar measurements as will be done around cloud base. However, the cloud optical thickness being much larger at this level than at cloud base, the feasibility of the method remains an open question at this particular level (it will be tested using simulators). Therefore, several alternative methodologies will also be considered.

One will consist in analysing the vertical distributions of the lidar backscatter signal and radar reflectivities measured from the downward-looking instruments on HALO and the upward and downward-looking instruments on the ATR-42. Another one will consist in analysing data from ground or ship-borne instruments. Ceilometers will be very useful, but a scanning radar (Oue et al. 2016) on one of the research vessels or deployed on Barbados is also being considered. Yet another approach will consist of analysing observations from the SpecMACs instrument on HALO. SpecMACs is a hyper-spectral line-imager with a field of view of about $40^{\circ }$ which allows to map a 10 km swath with 10 m resolution, this swath being similar to the anticipated one for the sideways staring lidar on-board the ATR-42. Oxygen A-band measurements from SpecMACS can be used to measure the distance to the cloud top, as can measurements from the thermal imager which is being developed for EUREC^4^A. Finally, satellite measurements such as those from lidar (either from CALIPSO (Winker et al. 2003) if it still operates in 2020, or from ADM-Aeolus and/or EarthCARE, that will be launched in 2017 and 2019, respectively) or high-resolution spectrometers such as ASTER, which has a 15-m horizontal resolution and many channels in the infrared, visible and near-infrared (Zhao and Di Girolamo 2007), will provide independent estimates of the vertical profile of cloud fraction.

The dynamic properties of clouds will be inferred from radar measurements (e.g., vertical velocities at cloud base will help determine whether a cloud is active or passive), and the microphysical properties will be derived from the combined analysis of radar-lidar measurements, passive radiometers and in situ measurements (Sect. 5.2). Vertically integrated cloud liquid content of shallow clouds will be measured using downward-looking radiometers flown aboard HALO. The occurrence of precipitation and mesoscale organization of precipitating shallow clouds will be characterized from the scanning precipitation (S-band) weather radar on Barbados and may be complemented by a scanning C-band research radar system.

Finally, measuring the radiative effects of clouds will be critical to assess the coupling between clouds and their large-scale environment. Vertically integrated estimates will be derived from broadband radiative fluxes measured near the surface, near the inversion level and in the upper troposphere, and vertical profiles of the radiative heating rate will be inferred from radiative transfer calculations using observed atmospheric and cloud properties.

### Inferring the Convective Mass Flux at the Top of the Subcloud Layer

To test the hypothesis that lower-tropospheric mixing critically influences the trade-cumulus cloud fraction at cloud base (e. g., Rieck et al. 2012; Gettelman et al. 2012; Sherwood et al. 2014; Brient et al. 2016; Vial et al. 2016), we will need to measure the strength of the convective mixing or quantities closely related to it. Indirect measures of convective mixing, for instance in terms of the relative humidity profile or the strength of shallow overturning circulations, are straightforward to infer from the large-scale structure of humidity field, and measurements of the large-scale vertical velocity. During EUREC^4^A a direct measure of convective mixing will also be provided in terms of the area-averaged mass flux at, or near, cloud base, *M*. Two independent methods will be used to estimate *M*: one based on direct measurements of the fractional area covered by active clouds and of the vertical velocity within them; the other based on the mass budget of the subcloud layer.

**Fig. 8:**
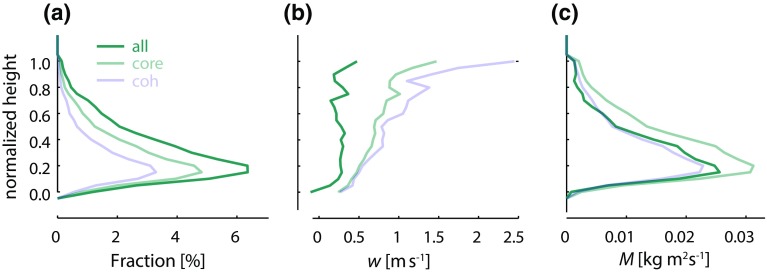
Boundary-layer profiles (normalized by the maximum cloud top height and minimum cloud base height) of hourly averaged **a** cloud fraction, **b** vertical velocity and **c** mass flux for all, core and vertically coherent updraft samples collected at the island of Graciosa in the Azores. (Adapted from Ghate et al. 2011)

The first method is an intuitive approach and will be executed by using radar measurements from the ATR-42 flying just above cloud base. Based on vertical velocity measurements within clouds ($w_{\mathrm {cld}}$), the fractional area covered by active clouds ($a_{\mathrm {cld}}$) will be measured. The cloudy mass flux, just above cloud base, will then be estimated as: $M = \rho a_{\mathrm {cld}}\cdot w_{\mathrm {cld}},$ where $\rho$ is the density of the air. A similar method has been applied to ground-based remote sensing measurements in the past (e.g., Kollias and Albrecht 2010; Ghate et al. 2011; Lamer et al. 2015; Ghate et al. 2016). Using an aircraft to make the same measurement greatly increases the sampling statistics, as in a given amount of time the aircraft samples many more (ten to fifteen times) cloudy updrafts. Using this method, the cloudy area (as well as properties within it) can be decomposed further into updraft and downdraft areas, or even further into different cloud parts (e.g., core or vertically coherent updraft, Fig. 8), or into a spectrum of cloud sizes (Neggers 2015). Similar and ongoing measurements of *M* from the cloud radar and wind lidars at the Barbados Cloud Observatory will provide additional context for these measurements, as during EUREC^4^A it is intended to target air masses upwind of the observatory.

The second method estimates *M* as a residual of the subcloud layer mass budget, whereby

3 $$\begin{aligned} \frac{{\mathrm {D}}\eta }{\mathrm {D}t} = E + W - \frac{M}{\rho }. \end{aligned}$$

**Fig. 9:**
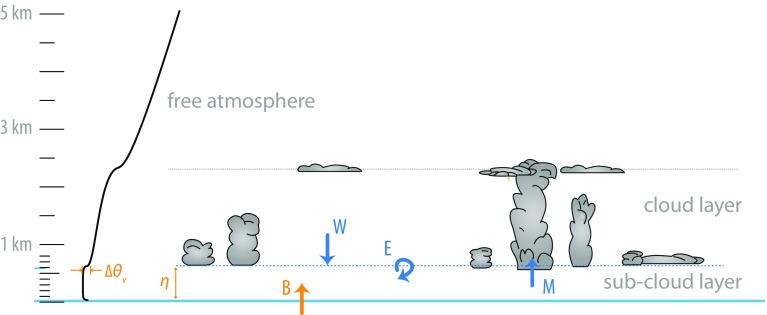
Schematic representation of the subcloud layer and of the main physical processes affecting its mass budget

Here $\eta$ denotes the depth of the subcloud layer, *E* is the top entrainment velocity, *W* is the large-scale vertical velocity at $\eta$ (which is related to $\omega$ at the same level by a coordinate tranformation) and *M* is the convective mass flux out of the subcloud layer. This budget is illustrated in Fig. 9.

To estimate *M* from Eq. (3) thus requires measurements of the other terms. *W* will be measured using the divergence methods discussed previously. All that remains is to estimate ${\mathrm {D}}\eta /{\mathrm {D}}t$ and *E*. The former, the substantial derivative of the subcloud layer depth, can be derived from a combination of soundings and downward-staring lidar aboard HALO, as this will provide both the time evolution of $\eta$ and the advective contributions to the substantial derivative.

The entrainment velocity, *E*,  will be estimated in a number of ways. One approach is to assume that the entrainment dynamics of the subcloud layer is the same as for the cloud free convective boundary layer, as appears to be the case for LES (Siebesma and Cuijpers 1995; Siebesma et al. 2003; Stevens 2006). *E* can then be diagnosed with the help of the buoyancy flux closure (Lilly 1968), which states that the buoyancy (equivalently virtual potential temperature) flux at $\eta$ is proportional to its flux at the surface: $(\overline{w'\theta _{\mathrm {v}}'})_\eta = -A(\overline{w'\theta _{\mathrm {v}}})_{\mathrm {s}},$ with *A* a proportionality constant of about 0.4 (Naumann et al. 2017). This allows *E* to be estimated from the flux-jump relationship at $\eta$ (Stevens 2006) as

4 $$\begin{aligned} E = \frac{A(\overline{w'\theta _{\mathrm {v}}'})_{\mathrm {s}}}{{\Delta }\theta _{\mathrm {v}}} \end{aligned}$$

where ${\Delta }\theta _v,$ is the jump in the virtual potential temperature across $\eta .$ For this calculation, $(\overline{w'\theta _{\mathrm {v}}'})_{\mathrm {s}}$ can be constructed from measurements of surface sensible and latent heat fluxes. To account for the thickness of the interfacial layer at $\eta ,$ the proportionality constant, *A*,  the jump, ${\Delta }\theta _v$ and $\eta$ must be estimated consistently (Garcia and Mellado 2014; Naumann et al. 2017). We propose to do so by fitting the observations of $\theta _v$ (as diagnosed by soundings and flights in the subcloud layer) with values just above cloud base (where the ATR-42 will mostly be flying) to LES in a manner consistent (as per the LES) with the chosen value of *A*.

A preliminary analysis of large-eddy simulations representative of typical trade-cumulus conditions (Vogel et al. 2016) shows that estimating *M* in this manner agrees reasonably well (within 35% for the initial calculations) with the value that is diagnosed directly from model output of cloud-core vertical velocity and cloud-core area fraction. The quantitative consistency between both estimates is sensitive to the definition of $\eta$ in the LES (both the maximum gradient in total humidity or the local minimum in the vertical velocity variance close to cloud base are suitable definitions), and on how the buoyancy flux at the top of the subcloud layer relates to the surface buoyancy flux. By the time of the EUREC^4^A field campaign, the present method will be refined by defining $\eta$ such that the estimated and diagnosed mass fluxes are in closer agreement ($\eta$ can be defined in several ways), and by accounting for the small temporal fluctuations in $\eta$. We will also investigate how much the method can capture the sensitivity of the mass flux to different boundary conditions such as sea surface temperature, wind speed and the large-scale divergence *D*, which are associated with different precipitation fluxes and different degrees of convective organization.

The entrainment velocity, *E*,  and hence *M*,  can also be diagnosed from tracer budgets. In principle, each independent tracer provides the basis for an independent estimate of *E*. As an example, the budget of the subcloud layer averaged equivalent potential temperature, $\overline{\theta _{\mathrm {e}}},$ takes the form

5 $$\begin{aligned} \frac{{\mathrm {D}} \overline{\theta _{\mathrm {e}}}}{{\mathrm {D}}t} = Q_{\mathrm {e}} + \frac{(\overline{w'\theta _{\mathrm {e}}'})_{\mathrm {s}} - E {\Delta }\theta _{\mathrm {e}}}{\eta } \end{aligned}$$

where $Q_{\mathrm {e}}$ is the radiative source/sink, and $(\overline{w'\theta _{\mathrm {e}}'})_{\mathrm {s}}$ the surface flux, of $\theta _{\mathrm {e}}.$ Given measurements of all the other terms, thus yields *E*. Most of the terms can be measured using methods similar to those already discussed above. Irradiances, which are required to estimate $Q_{\mathrm {e}},$ will be measured directly (along the near-surface and above boundary-layer legs of the ATR-42), but also estimated on the basis of radiative transfer calculations given the atmospheric state. A similar approach can, and will, be adopted for estimating *E* from the water budget, which will then require estimates of the precipitation rate at $\eta$ and at the surface. Taken together with estimates based on Eq.(4) results in three different methods for estimating *E*,  and hence inferring *M* as a residual of Eq. (3).

The comparison of the direct and budget methods for estimating *M* will help to assess the robustness of the estimates, especially regarding the sensitivity of the mass flux variations to changes in environmental conditions. This assessment will also test understanding of the subcloud layer budget, most importantly the extent to which *M* at the top of the subcloud layers as defined in Eq. (3) is related to *M* a short distance above cloud base and as used to parameterize cumulus convection, an equivalence that should not be taken for granted.

## A Benchmark Data set

Previous reference observational data sets for linking clouds to circulation in the trades are those from BOMEX, ATEX and GATE. These are field studies which took place nearly a half century ago before the advent of satellite remote sensing, not to mention transformative progress in simulation science. Through a close integration with satellite remote sensing and advances in modelling, EUREC^4^A aims to provide a reference data set for studying clouds and circulation in the trade-wind region.

### A Simulation and Modelling Testbed

Large-eddy models have long been used to simulate trade-cumuli. Nowdays, they are run over increasingly larger domains, which allows shallow convection to organize into spatial patterns on the mesoscale (e.g., Seifert and Heus 2013; Vogel et al. 2016). The apparent realism of the circulations and clouds that develop often encourages their adoption as an adequate description of reality. However, LES incorporates approximations and assumptions in addition to those associated with the choice of boundary forcings for the simulation. These include the numerical methods adopted, which are known to significantly affect cloud structure and fraction (Vial et al. 2017), as well as the way in which radiative transfer, cloud microphysics and small-scale turbulent motions are parameterized. Most of the LES evaluations of cloud fields have been using observational data from ATEX, BOMEX or RICO (Stevens et al. 2001; Siebesma et al. 2003; vanZanten et al. 2011). These observations make it possible to evaluate carefully the thermodynamic structure of the boundary layer for a limited set of given large-scale forcings. However they do not answer critical questions such as: What is the typical cloud cover and what is the fraction of the cloudy air that is positively buoyant? How strong is the cumulus mass flux at cloud base? How does the cloud fraction and cloud water content vary with changes in the large-scale environment? By measuring important properties of the cumulus mass flux, the large-scale vertical velocity and the large-scale environment, the EUREC^4^A campaign will offer opportunities to answer some of the above-mentioned questions and to critically test the fidelity of large-eddy simulations.

One process of particular interest, is the “cumulus-valve mechanism” for regulating cloud-base mass fluxes (e.g., Neggers 2015). This mechanism suggests that the mass flux is that required to maintain the cloud-base cloud fraction nearly constant. During EUREC^4^A, as vertical velocities within clouds will be measured by radar measurements, we will evaluate to what extent it is operative, and the degree to which this indeed controls cloud base cloud fraction. Another question is to what extent mesoscale variability, which may often be the “flow-debris” of much larger-scale circulations not represented by LES, is important for determining cloudiness and its variability. For instance, the influence that cold pools or surface temperature heterogeneities associated with submesoscale processes in the ocean (Sect. 5.6), may exert on cloudiness remains an open issue.

By computing large-scale forcings (water vapour and heat large-scale advections) from EUREC^4^A observations, it will also be possible to run single-column versions of large-scale models (Single-Column Models or SCMs). It will help us to test the model physics further, and also better understand the cloud feedbacks produced by these models. Indeed, there is ample evidence that single-column simulations of shallow cumuli can help understand low-cloud feedback processes and their dependence on process representations (e.g., Brient and Bony 2012, 2013; Zhang and Bretherton 2008; Zhang et al. 2013a; Dal Gesso et al. 2015; Brient et al. 2016). The link to observations, and the comparison between LES and SCM simulations, will allow us to investigate the relationship between the response of shallow cumuli to prescribed climate change perturbations and the realism of the simulated clouds in the present-day climate, which will help answer questions such as: How does the cloud cover depend on the strength of convective mixing? How variable is it with changes in environmental conditions? Is it possible to constrain the strength of climate change low-cloud feedbacks from present-day processes? (Vial et al., this volume). Taken all together, the new experimental methodologies being developed and deployed as part of EUREC^4^A will provide new opportunities to provide a reference data set to inform modelling and simulation of trade-cumuli. Besides the evaluation of shallow clouds and cloud feedback processes, it will also help us evaluate the representation of physical processes in climate and weather models, including the parameterization of cumulus convection in large-scale models and the high-resolution operational forecast models used to predict weather in the trades (Sect. 5).

### A Remote Sensing Testbed

Observations from field campaigns are not only fundamental to investigate the physics of trade-cumuli but also to test, and eventually improve, the instruments and algorithms of remote sensing that are used to observe the Earth from space. Beyond the evaluation of cloud retrievals from current satellites, EUREC^4^A is expected to contribute to the evaluation of the cloud and wind retrievals from two new flagship satellite missions of the European Space Agency: ADM-Aeolus and EarthCARE, that will provide unprecedented information on clouds and circulation.

During EUREC^4^A, the instruments on-board HALO and the ATR-42 aircraft will sample almost the full spectrum of wavelengths of atmospheric electromagnetic radiation (from the UV to the microwave), making it possible to retrieve a wide range of geophysical properties. Moreover, the UV HSR Doppler wind lidar operating at 355-nm and the 95 GHz Doppler cloud radar will provide products comparable to those obtained from the ADM-Aeolus and EarthCARE satellites. When flying underneath the satellite orbits, it will thus be possible to make direct comparisons between airborne and space-borne measurements. The in situ observations will help interpret the remote sensing in terms of geophysical variables, and the comparison between airborne and space-borne measurements will help evaluate some of the limitations of the satellite remote sensing.

The main limitations of remote sensing are due to the lack of sensitivity of the sensors (which is of particular concern when probing the lower atmosphere from space, but much less from an aircraft), the inability to exploit some measurements near the surface (e.g., the blind zone arising from the contamination of radar measurements by ground clutter or from the saturation of the lidar signal) and the poor spatial resolution of the measurements (which is particularly problematic in areas covered by small broken clouds such as shallow cumulus fields). For passive measurements, which have the best spatial coverage, a particular challenge is identifying sufficiently unique information to deconvolve the atmospheric vertical structure from signals that necessarily integrate over this structure. The synergy of in situ, airborne and space-borne measurements during EUREC^4^A, jointly with high-resolution simulations from weather forecast models and LES simulations of the campaign area, will help quantify these different sources of uncertainty and test some of the hypotheses used in the cloud or wind retrieval algorithms. A few examples are given below.

Satellite instruments like MODIS or MetOP measure radiances in different wavelength channels, and these measurements are used to retrieve cloud droplet number, cloud phase, optical thickness, and droplet or particle size at cloud top at a spatial resolution of about 1 km. This resolution is insufficient for the observation of shallow cumuli. The specMACS instrument on-board HALO (Appendix 1), which combines hyper-spectral wavelength resolution in the visible and near-infrared wavelength range with a spatial resolution of about 10 m, will permit to observe trade-wind clouds in greater detail. Obvious products will be cloud cover and cloud-size distributions. In addition, the use of three-dimensional radiative transfer methods will permit to retrieve cloud optical thickness, droplet radius, and cloud top structure with high spatial resolution (Mayer 2009; Zinner et al. 2006) and may even be able to distinguish the cloud-core area from the optically thinner edges. The contribution of clouds to solar heating and infrared cooling rates will also be estimated from these parameters.

Another key property of clouds for which satellite retrievals remain very uncertain is the cloud liquid water path (LWP). Most of today’s knowledge on the global distribution of cloud liquid water is derived from polar orbiting satellites that measure radiances in the thermal infrared and microwave spectral regions. However, given the coarse spatial resolution of these measurements (several tens of kilometres), the LWP retrievals critically depend on the estimated cloud fraction (Horváth and Gentemann 2007) and cloud vertical structure (Borg and Bennartz 2007). The synergy of the HALO and ATR-42 instrumentation will provide fine-scale information on the variability of water vapour, liquid water and cloudiness over an area of $200 \times 200 \, \hbox {km}$ which will help evaluate satellite retrievals and the validity of their underlying assumptions. By combining active (radar) and passive microwave radiometer, it may also be possible to quantify the amount of drizzle and precipitation in fields, or aggregates, of shallow cumulus. Beyond its intrinsic interest, this detection will make it possible to test the validity of the precipitation thresholds used in LWP retrievals (Wentz and Spencer 1998), and thus to improve LWP retrievals in trade-wind regions.

Active satellite remote sensing provides observations along narrow curtains aligned with the flight track. To achieve radiative closure (as envisioned with EarthCARE) or to generate precipitation fields (as done as part of the GPM mission), it is crucial to combine curtain measurements with observations from wide swath instruments. For this purpose, LES simulations are often used to get statistical information about the three-dimensional structure of the cloudy atmosphere. By providing the reference data set necessary to assess such statistics (Sect. 4.1), EUREC^4^A will thus help assess and improve these techniques, and may also help guide future satellite measurements to, for instance, better profile lower-tropospheric water vapour.

Finally, the LNG (lidar) instrument on-board the ATR-42 will have the capability to mimic ADM-Aeolus measurements: the 355-nm HSR Doppler wind lidar of the satellite will point 35 deg from nadir (orthogonal to the ground track velocity vector to avoid contribution from the satellite velocity) to derive profiles of the horizontal wind component, and will also regularly point to nadir for calibration. LNG measurements along the satellite orbits and in the same viewing direction, together with in situ measurements and other airborne observations, will help evaluate the L2A (cloud and aerosol optical properties) and L2B (radial winds) ADM-Aeolus products over distances of several hundreds of kilometres.

## EUREC^4^A^++^: An Opportunity for Complementary Investigations

The intensive observations of the atmosphere and of the surface that will be collected during EUREC^4^A campaign will provide an opportunity to address additional scientific issues. A few of them are mentioned below in the context of what we call EUREC^4^A^++^, but more are almost certain to arise in the next years.

### Rectification of Large-Scale Vertical Motions by the Diurnal Cycle of Shallow Clouds

In addition to providing large-scale context for the aircraft missions, measurements by the large-scale sounding array will supplement (and test) meteorological analyses to help answer scientific questions such as: What is the role of transient disturbances and their influence over large-scale vertical motion in modulating convective mass flux and large-scale diabatic heating? What drives the diurnal cycle of vertical motion and clouds in the trades?

Mounting evidence indicates that the Hadley cell over the remote oceans is characterized by a pronounced diurnal cycle in overturning motion, quite distinct from the influences of land (Nitta and Esbensen 1974a; Gille et al. 2003; Wood et al. 2009). It is possible that this diurnal cycle owes fundamentally to the response of deep convective clouds in the ITCZ to the diurnal cycle of direct shortwave absorption (Nitta and Esbensen 1974a), although this topic is unresolved. Observations from suppressed regimes in the Indian Ocean warm pool region reveal that the diurnal cycle in large-scale vertical motion is intimately tied to a diurnal cycle in the shallow convective-cloud population: clouds deepen each afternoon as subsidence relaxes, while the afternoon increase in more active, precipitating clouds leads to more cold pools that in turn augment cloud area fraction (Ruppert and Johnson 2015, 2016). Experiments conducted with a LES framework suggest that this diurnal cloud feedback between large-scale vertical motion and macroscopic cloud properties augments diabatic heating, thus impacting large-scale circulation, on longer timescales through nonlinear rectification (Ruppert 2016).

An additional objective of EUREC^4^A will therefore be to diagnose the relationships between radiation, clouds, and large-scale vertical motion on the diurnal timescale, and to relate this timescale to other modes of variability. The target hypothesis pertaining to this process is that the diurnal shortwave heating cycle drives a diurnal cycle of deep convection in the ITCZ, which in turn drives a diurnal cycle of large-scale overturning motion in the greater Hadley cell.

### Cloud Microphysics

Cloud macrophysical properties such as cloud fraction or cloud water content are very much influenced by the large-scale environment (e.g., the strength of large-scale vertical motion, tropospheric subsidence, surface temperature, tropospheric humidity). In this context, unravelling the impact of microphysical processes on cloud macrophysical properties or radiative fluxes is challenging because microphysics, cloud macrophysics and large-scale environmental conditions vary in concert. Given that the large-scale dynamical and thermodynamical conditions, and cloud macrophysical properties, will be quantified by EUREC^4^A, it will be easier to assess the impact of microphysics on cloud macrophysics for given large-scale conditions, and to explore the dependence of microphysics on large-scale environmental conditions or convective organization. For instance, we might investigate whether in observations, precipitation affects the growth of shallow cumuli as LES studies suggest (Vogel et al. 2016).

EUREC^4^A will offer several opportunities for microphysical measurements: besides in situ liquid and total water content, cloud droplet size distribution and particle imaging on-board the ATR-42 (Table 1), aerosol measurements at the BCO may also be supplemented by measurements from research vessels. In situ data could be collected by small autonomous vehicles launched from the ships or the island (e.g., drones) as well as a tethered HeliKite capable of carrying payloads of up to $100 \, \hbox {kg}$ to heights of $3 \, \hbox {km}$. The possible deployment of additional aircraft (from the UK and US), that would focus on microphysical measurements, is also being considered. Such measurements would greatly help advance understanding of cloud-aerosol interactions, entrainment and mixing processes and the onset of precipitation within shallow clouds.

### Shallow Clouds and Convective Momentum Transport

Concurrent measurements of clouds and the large-scale horizontal wind profile during EUREC^4^A will also help address open and long-standing questions regarding the two-way interaction of clouds (convection) and winds. Past studies have investigated which parameters that represent the large-scale atmospheric state may best predict low-level cloud amount (sometimes referred to as “cloud-controlling” factors). Although this is challenging within the trade-wind cumulus regime—there is no single strong predictor, and correlations on timescales less than a month are small—the correlation between low cloud amount and the near-surface wind speed appears one of the stronger correlations (Brueck et al. 2015; Nuijens et al. 2015b). Daily surface wind speeds have also been found to correlate well with daily averaged rain cover.

This relationship may reflect the influence of surface wind speed on the surface enthalpy fluxes, and hence the depth of convection and the depth of the trade-wind layer. But the relationship may not just represent a one-way interaction: clouds themselves also influence the wind profile through convective momentum transport. This transport would alter winds across a much deeper layer than dry convection and turbulence in the subcloud layer can do. Depending on the wind profile beyond the subcloud layer, convective momentum transport may therefore slow down or accelerate winds near the surface through processes which may also depend on the degree of mesoscale organization of the cloud field. The wind measurements made during EUREC^4^A will help investigate how winds influence clouds and vice-versa. The opportunity to enhance these measurements by using two different wind lidar systems as part of a funded ADM-Aeolus validation mission using DLR’s Falcon aircraft is being considered.

### Improving Climate and Weather Forecast Models

In addition to helping evaluate the processes that control trade-cumuli and cloud feedbacks in LES and GCM models (Sect. 4.1), EUREC^4^A will help evaluate more generally the physics of large-scale climate and weather models. Indeed these models still exhibit significant biases in the representation of clouds and circulation in the trades. For instance, most of them overestimate the reflection of solar radiation by trade-cumuli despite an underestimate of the cloud fraction and/or the cloud water (the so-called too few, too bright problem, Karlsson et al. 2008; Nam et al. 2012). Models also exhibit persistent biases in their simulation of the surface wind stress (Wang and Carton 2003; Simpson et al. 2014) which, as discussed above, can relate to wrong representations of the surface drag and/or of the momentum transport by shallow convective clouds (Polichtchouk and Shepherd 2016; Schlemmer et al. 2017). The comparison with EUREC^4^A observations of short-term forecasts run with such models will help disentangle sources of model errors in the representation of physical processes and their interaction with the large-scale circulation. Moreover, as discussed in Sect. 3.3, a better understanding and assessment of the different contributions to the subcloud layer energy budget will help assess the hypotheses underlying the cumulus mass flux closures used in convective parameterizations.

More specifically, EUREC^4^A will serve as a testbed for high-resolution modelling approaches developed by the Caribbean Institute of Meteorology and Hydrology, Meteo-France, KNMI and ECMWF to deliver operational forecasts to the Caribbean countries. Improving the quality of these forecasts is critical, especially regarding high-impact weather. In Barbados for instance, heavy precipitation produced by severe weather frequently produce significant flash flooding. Landslides, particularly on neighbouring islands with greater orographic relief, result in significant social and economic losses including loss of property and livelihoods and on occasion loss of life. Losses from such events can range from 25–200% of national Gross Domestic Product setting back national development by more than a decade in some instances. In order to reduce losses, in recent years there has been a significant effort to improve early warning hydro-meteorological forecasts over the Caribbean through the development of high-resolution (4 km) numerical weather forecasts using the Advance Research Weather Research and Forecasting (WRF) model. By providing a reference data set for the evaluation of cloud, turbulence and convective parameterizations, EUREC^4^A will help improve the physics of the model, and eventually the quality of the rainfall forecasts.

### Improving Model Convective Parameterizations

Another important objective of the experiment is to provide field experimental data by which new and existing convective parameterizations can be tested. Measurements of mass fluxes during EUREC^4^A can be used to test closure assumptions, as discussed, but measurements of convective fluxes would provide an opportunity to test how the convective scheme distributes the energy it carries across cloud base.

We know from many observations that the vertical enthalpy flux establishes a temperature lapse rate that is very close to moist adiabatic, and most existing schemes are designed to accomplish this. For this reason, comparing the parameterized enthalpy flux against observations is a weak test of performance. On the other hand, there is no corresponding universal water vapour profile, and it can be shown that the vertical flux of water depends on such processes as entrainment and cloud microphysics. For this reason, the vertical subgrid-scale water flux by a convection scheme also presents an opportunity for a strong test of convective schemes.

Measuring the vertical flux of water in a field programme is extremely challenging, as the decisive component is the small residual between large, but opposed, vertical turbulence fluxes and those by precipitation. During EUREC^4^A, the density of the sondes, and the availability of water vapour lidar profiling, and the variety of radar products for estimating precipitation, should provide an excellent opportunity to constrain the water budget, perhaps to a degree that it can help test convective parameterizations. Fortunately, in the Tropics, fluctuations of moist static energy are strongly dominated by fluctuations in water vapour, because temperature perturbations are usually very small above the boundary layer. Thus, the moist static energy budget also offers and opportunity for deducing the convective flux of water vapour, principally by estimating the terms in the budget, or partially integrating measurements of the budget from the top of the atmosphere (or the surface) to the flight level, and then estimating the convective flux as a residual.

Note that it will be possible to assess the overall quality of the field measurements needed to test convective schemes by verifying that:

6 $$\begin{aligned} \frac{1}{g}\int _0^{P_{\mathrm {s}}} \left( \frac{\partial h}{\partial t} + {\mathbf {V}}\cdot \nabla h\right) \, {\mathrm {d}}P + F_{\mathrm {TOA}} - F_{\mathrm {SFC}} = 0 \end{aligned}$$

where *h* is the moist static energy, ${\mathbf {V}}$ the three-dimensional wind, $F_{\mathrm {TOA}}$ the net radiative flux at the top of the atmosphere and $F_{\mathrm {SFC}}$ the total (radiative plus turbulent) surface energy flux. In previous field experiments, errors in some of the terms in (6) resulted in a nonzero sum, which had to be corrected by making adjustments to the vertical velocity used in the advection term (Emanuel and Zivkovic-Rothman 1999). Hopefully, these undesirable errors can be largely avoided in EUREC^4^A by obtaining more accurate estimates of vertical velocity as well as improved estimates of radiative and surface energy fluxes. By providing more accurate field data sets—ones satisfying column water and moist static energy budgets—more rigorous tests of cumulus parameterizations should be possible. During EUREC^4^A, these methods will be tested for regimes of shallow convection. If successful they could later be applied in regions of deep convection.

### Ocean Eddies

EUREC^4^A will also be an opportunity to study ocean-atmosphere interactions in the Atlantic, especially the role of ocean mesoscale eddies. The ocean is a fundamentally turbulent fluid full of fine-scale structures such as eddies, fronts, jets and filaments (McWilliams 2016). These oceanic structures, grouped as mesoscale (10–500 km, 10–100 days) and submesoscale (hundreds of metres to kilometres, daily timescales) dynamics, are recognized as key contributors to the ocean circulation (e.g., Zhang et al. 2013b). There is also increasing evidence that they impact air–sea interactions and influence the winds and clouds of the overlying atmosphere (Chelton et al. 2004; Ferreira and Frankignoul 2008; Frenger et al. 2013; Byrne et al. 2015). However, few observations are available to quantify the role of ocean eddies in the transport of water properties and in air–sea interactions, especially in the tropics.

**Fig. 10:**
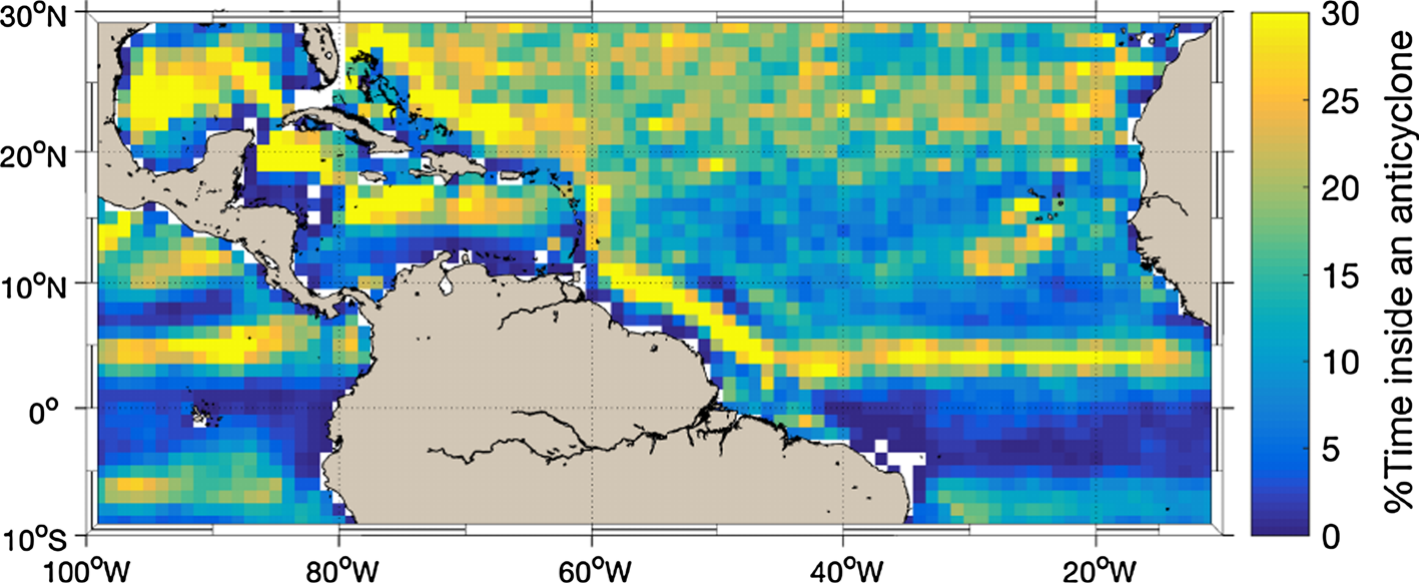
The route of ocean eddies. Statistics of ocean mesoscale eddies derived from satellite altimetry (shown is the fraction of the time inside an anticyclonic eddy)

Automatic eddy detection from satellite observations show the presence of mesoscale eddies (Fig. 10) not only in the mid- and higher latitudes but also in the Tropics (Laxenaire et al. 2017, accepted). In particular, intense warm ocean eddies (i.e. anticyclonic eddies) converge in the western tropical Atlantic, offshore of Barbados. These eddies come from the south (tropical Atlantic and South Atlantic) and from the East (from Cape Verde and the western Africa margin). Eddies such as the anticyclonic features associated with North Brazil Current Rings eventually carry freshwater, originating from the Amazon/Orinoco river, into the region. Linkages between the freshwater surplus by the rivers and intensification of storms and cyclones have been reported for the large scale (Reul et al. 2014) and for the region east of the Antilles, in the Caribbean, for individual eddies (Rudzin et al. 2017).

**Fig. 11:**
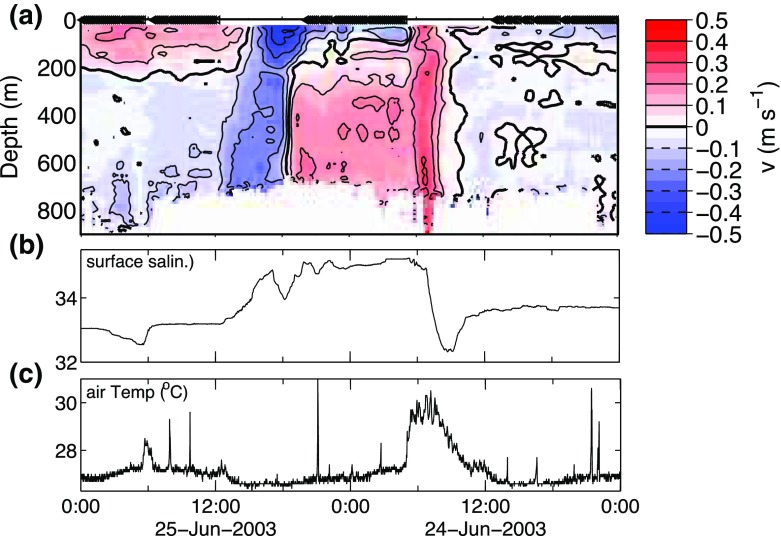
RV SONNE (SO172) ship survey through an anticyclonic eddy (North Brazil Current Ring) west of Barbados (ship was going east to west, time axis is reversed for clarity). **a** Meridional current section (triangles at 0 m denote ship was stationary), **b** sea surface salinity (psu), and **c** air temperature

Inspecting historical ship data in the EUREC^4^A campaign area (RV SONNE SO172; Fig. 11) shows the impact of low salinity water in an anticyclonic North Brazil Current Ring that is associated with increased air temperature and the occurrence of very local air/sea interactions. During the EUREC^4^A campaign observations by aircraft, research vessels and autonomous platforms will make it possible to observe the coupled system and assess the role of mesoscale eddies on the lower tropical atmosphere in a comprehensive manner. In particular, the association of air and ship measurements will allow us to characterize contrasted ocean mesoscale eddies (e.g., cyclonic/anticyclonic, deep/shallow mixing) estimate the role of these eddies in modifying atmosphere-ocean coupling and in influencing the mesoscale organization of the atmosphere and shallow clouds in particular.

Moreover, a synoptical study from different research vessels measuring different mesoscale eddies across the experimental area will provide new information on water-mass characteristics advected by the regional eddies. These are of twofold importance, as they inform studies of mesoscale eddies in general, but also in a particularly interesting region where over a narrow zone of longitudes the upper and lower limbs of the Atlantic Meridional Overturning Circulation flow over one another. A relatively wide range of mesoscale eddy observations will provide quantitative assessments of their role in transport of mass, heat, freshwater, carbon and other biogeochemical variables enabling a measure of the role of the oceanic mesoscale in this overturning circulation.

The observing strategy will make use of near real-time satellite altimetry data (AVISO Ssalto/Duacs) to identify ocean mesoscale eddies and then one of the ships will be used to survey the eddy and to deploy autonomous oceanographic observing platforms such as underwater electric gliders and wave-gliders. A second ship will characterize the surrounding background field in parallel. Vertical profiling of the water column (temperature, salinity, currents, oxygen, and other properties e.g., carbon related quantities) will be measured. Argo profiling floats will be deployed within anticyclonic eddies within the Guiana and North Equatorial currents in the year preceding the EUREC^4^A campaign. More information about the instrumentation and the measurements envisioned is given in Appendix 2. This strategy will be tested and refined before the campaign by undertaking a set of preliminary studies based on the analysis of available data from satellites, ships, Argo profiling floats, and eddy-resolving numerical simulations.

Together, and perhaps the biggest plus, is that oceanic and atmospheric data collected during EUREC^4^A will help build a data set capable of evaluating a wholly new generation of coupled ocean-atmosphere models, ones capable of resolving (rather than parameterizing) both convective eddies (cumulus convection) in the atmosphere, and the mesoscale dynamics of the ocean.

### Capacity Building

Building national and regional resilience to increasing climate variability, climate change and extreme weather events in the Caribbean includes increasing national and regional weather and climate data, related knowledge platforms and human capacity. By involving university students and other young scientists of the region in the field campaign and the long-term research activities related to it, EUREC^4^A will help train the next generation of regional climate scientists and operational forecasters, develop databases that will facilitate the dissemination and use of the data collected in the area during field studies, and will promote international partnership and collaboration networks.

## Conclusions

By characterizing, for the first time, both the macrophysical properties of shallow cumuli and the large-scale environment in which convection and clouds are embedded, the EUREC^4^A campaign will test developing ideas about what controls the cloud amount in the trades. It should elucidate the role of convection and large-scale circulations in low-cloud feedbacks and thus address one of the central questions of the World Climate Research Programme’s Grand Challenge on *Clouds, Circulation and Climate Sensitivity* (Bony et al. 2015). Through its alignment with two flagship missions of the European Space Agency and the cutting-edge of modelling, EUREC^4^A should also provide a new reference data set which can be used to assess the modelling and the remote sensing for the years to come. The experimental strategy proposed for the campaign is ambitious. However, it builds on a legacy of ongoing field studies, particularly ground measurements at the Barbados Cloud Observatory and field measurements as part of the NARVAL (December 2013) and NARVAL2 (August 2016) campaigns, as well as extensive experience from process models. Ongoing analysis of these measurements and simulations are being used to test and refine the experimental strategy of EUREC^4^A so as to maximize the scientific gains from the planned measurements.

A compact and well-defined experimental strategy opens up the mission to other partners with complementary interests. This campaign should therefore be considered as an opportunity to nucleate larger international efforts and to underpin additional investigations ranging from factors influencing cloud microstructure and warm rain formation, to the role of mesoscale eddies in the ocean.

## Appendix 1: Aircraft Instrumentation

Airborne platforms are one means to probe the thermodynamic, dynamic and cloud properties of the atmosphere. EUREC^4^A will be centred around measurements from two aircraft, namely the French ATR-42 and the German HALO, carrying complementary payloads. The HALO measurements aloft will characterize with downward-looking instruments the large-scale (several thousands of km^2^) environment in which clouds form, and the radiative properties of the clouds therein. The ATR-42 measurements in the shallow cloud layer will constrain cloud macrophysical and microphysical properties, turbulent mixing processes and shallow circulations.

The airborne and in situ measurements of EUREC^4^A will also advance the evaluation and calibration of space-borne measurements, and their interpretation in terms of geophysical variables (Wendisch and Brenguier 2013). It will be the case in particular for observations from EarthCare and ADM-Aeolus, which are two flagship satellite missions of the European Space Agency’s Living Planet Programme devoted to the observation of clouds and circulation, and whose measurements will be mimicked by several instruments on board both aircraft.

### The ATR-42 Aircraft and Its Instrumentation

The French ATR-42 is a bi turbo-prop aircraft from SAFIRE that has the capability of flying in the lower troposphere (ceiling at about 8 km) with a maximum range of about 1800 km. It will fly a series of low-level legs just above cloud base (around 1 km), about 100 km long and spaced by about 20 km, as a way to sample the cloud field within the area encompassed by HALO circles. A particularity of the aircraft instrumentation (summarized in Table 1) is that it will include sideways and vertically pointing lidar and radars that will probe the atmosphere horizontally and vertically, aiming at measuring the cloud fraction at cloud base. The last leg before refuelling will be flown either below cloud level, to measure surface turbulent fluxes, temperature at the sea surface and in the subcloud layer, and near-surface radiation, or near the trade-inversion level (around 2 km) to characterize cloud microphysics, measure radiative fluxes. Given the mean science speed of the aircraft (about $100 \, \hbox {m}\,\hbox {s}^{-1}$) and the endurance expected for the envisioned payload, the ATR-42 will make two four-hr flights per day bracketed by the daily nine-hr flight of HALO.

The ATR-42 will be equipped with advanced instrumentation including the multi-wavelength Leandre New Generation (LNG) HSR backscatter lidar (Bruneau et al. 2015), the 95 GHz Doppler radar RASTA (RAdar SysTem Airborne, Delanoë et al. 2013), the mini-cloud radar BASTA (Delanoë et al. 2016), and the mini-lidar ALiAS developed at LSCE (Chazette et al. 2007; Chazette 2016). The first three instruments have been developed at LATMOS^1^ to characterize clouds, aerosol particles and hydrometeor particle velocities. Although RASTA and the HSR lidar will be observing the atmosphere above and below the aircraft, the mini-lidar ALiaS and the BASTA Doppler radar will be staring sideways from the aircraft windows in order to measure the cloud fraction and cloud optical properties just above cloud base (Sect. 3.2). In addition, the payload will include a thermal infrared radiometer (CLIMAT-AV, Conveyable Low-Noise Infrared Radiometer for Measurements of Atmosphere and Ground Surface Targets -Airborne Version) developed at the Laboratoire d’Optique Atmosphérique (Brogniez et al. 2003) to measure sea surface temperature, and hemispheric broadband pyrgeometer (Kipp and Zonen CGR4) and pyranometer (Kipp and Zonen CMP22) to measure upwelling and downwelling radiative fluxes in the longwave and shortwave, respectively. Finally, the aircraft will measure temperature, moisture and wind along its trajectory at a high frequency ($25 \, \hbox {s}^{-1}$) for turbulence statistics calculations.

The LNG airborne Lidar system is a three-wavelength (1064, 532, and 355 nm) backscatter lidar with polarization and high-spectral-resolution capability at 355 nm^2^. It operates in a direct detection mode (measurement of the backscattered light intensity), which has the advantage of relying on both particulate and molecular scattering, and allows extended ranges and capabilities. Thanks to its two-wave interferometry system (Mach–Zehnder Interferometer) and its capability of high-spectral-resolution UV analysis, it can determine both optical parameters of aerosol and clouds and measurements of along-line-of-sight wind velocity, based on the Doppler effect on particles (Bruneau and Pelon 2003; Bruneau et al. 2015). When scattering particles are moving, the wavelength of the scattered light is shifted by a small amount as a function of speed. The Doppler wind lidar measures this change of wavelength to determine the velocity of the wind in the direction of the light pulse. For a vertically pointing lidar, vertical velocity measurements are thus possible. The capability of a lidar system using a Mach–Zehnder interferometer is described in Bruneau and Pelon (2003) and is not be detailed here. A simplified equation describing the precision of LNG-derived wind velocity measurement along the line of sight can be obtained from Bruneau and Pelon (2003) (see their equation 23) as: $\sigma (V) \approx \frac{100}{SNR}\,\frac{R_\beta }{R_\beta - 1}$ (in $\hbox {m.s}^{ -1}$), where SNR is the signal-to-noise ratio on the 355-nm parallel channel and $R_\beta$ the backscatter ratio (i.e. the total backscatter divided by the molecular backscatter) measured on the same channel. The accuracy of the LNG system along the line of sight after aircraft motions are removed has been assessed for a case of cirrus clouds by Bruneau et al. (2015) based on the apparent aircraft speed derived from LNG ground-echo, and based on results from a recent field campaign (NAWDEX—North Atlantic Waveguide and Downstream Impact Experiment, fall 2016): the accuracy of LNG wind speed measurements in these conditions was expected to be around $1 \, \hbox {m s}^{-1}$ (D. Bruneau, personal communication).

The Airborne Lidar for Atmospheric Studies (ALiAS) was built at LSCE following a precursor instrument (Chazette et al. 2007; Chazette 2016) flown on-board an ultra-light aircraft (ULA). It is based on a frequency-tripled Nd:YAG laser (ULTRA) manufactured by QUANTEL^3^ emitting in the near ultraviolet (355 nm), thus satisfying eye safety requirements at the output window. The UV pulse energy is 30 mJ, and the pulse repetition rate is 20 Hz. The acquisition system is based on a PXI (PCI eXtensions for Instrumentation) technology with a sampling frequency of 200 MHz (initial resolution along the line of sight equal to 0.75 m). The receiver includes two channels for the detection of the elastic backscatter from the atmosphere in the parallel and perpendicular polarization planes relative to the linear polarization of the emitted radiation. It was designed to monitor both the aerosol and hydrometeor distributions and dispersions in the low and middle troposphere. After specific signal analysis, including laser shot accumulation and low pass filtering, the final resolution along the line of sight is between 15 and 30 m. With a 15-cm diameter telescope, the lidar is compact ($\sim \, 70 \times 45 \times 18 \, \hbox {cm}$), lightweight ($< 50 \, \hbox {kg}$ for both optics and electronics), robust to vibration, requires moderate power (< 500 W) and can thus be easily mounted aboard an aircraft for horizontal shooting. Its wide field of view (FOV) of 2.3 mrad ensures a full-overlap of the transmitter and receiver paths beyond 100 to 200 m.

The BASTA (Bistatic Radar System for Atmospheric Studies) radar is a mini-cloud W-band radar (weighting only 32 kg) that measures the Doppler velocity and the reflectivity at 95 GHz (Delanoë et al. 2016). Its specificity, compared to traditional pulsed radars, is that instead of transmitting a large amount of energy for a very short time period (as a pulse), a lower amount of energy is transmitted continuously. The radar can be used in several modes depending on application, including 12.5- and 25-m-vertical resolution modes. The sensitivity of the instrument is about -40 dBZ at 1 km for 3-s integration and a range-gate of 25 m. Its unambiguous range is 12 and 18 km for the 12.5- and 25-m range-gate modes, respectively. The high mobility of the system allows to install the system with an horizontal pointing configuration. The most will be made of the bistatic nature of the system, with emitting and receiving radar signals through two side-by-side windows. Calculations suggest that a cloud radar having a sensitivity of −35 dBZ at 1 km in an atmosphere with 80 % relative humidity will detect clouds with a reflectivity below −30 dBZ over 1400 m, and will not detect any liquid cloud further 3250 m. Considering a liquid cloud with a constant LWC of 0.6 g/m3 (which is a large upper bound) next to the aircraft, the radar should be able to detect clouds below −30 dBZ until 1000 m and liquid cloud until 2000 m.

The RASTA (RAdar SysTem Airborne) radar measures the Doppler velocity and the reflectivity at 95 GHz (W-band) along a radial defined by the pointing direction of the antenna (Delanoë et al. 2013). During EUREC^4^A, a 4-antenna configuration will be used, that will include 3 upward-looking beams (zenith, 28 degrees off-nadir perpendicular to the aircraft motion, and 20 degrees off-zenith and opposite the aircraft motion) and one nadir pointing antenna. This unique configuration allows for the retrieval of the three-dimensional wind field, i.e. the three components of the wind on vertical plan above the aircraft, by combining the independent measurements of the projected wind vector on radar line of sights. The independent Doppler radial velocities are provided by the multi-beam antenna system. The radar range is 15 km, its range resolution is 60 m and its horizontal resolution ranges from 100 to 150 m depending on aircraft speed. RASTA measurements of vertical velocities within clouds, combined with lidar-radar estimates of the cloud fraction at cloud base, will help develop an estimate of the convective mass flux at cloud base that will be completely independent of the one derived from the analysis of the subcloud layer mass budget (Sect. 3.3).

The CLIMAT-AV thermal infrared radiometer (Brogniez et al. 2003) measures (at nadir) radiances simultaneously in three narrowband channels centred at 8.7, 10.8, and 12.0 micron, with about 1 mm of full width at half maximum. It uses a 7-Hz sampling frequency and performs measurements within a 50-mrad field of view, which corresponds to a footprint of about 50 m at a 1-km range. CLIMAT-AV is very similar to the CALIPSO IIR system. The absolute accuracy of brightness temperature measurements is about of 0.1 K, and its sensitivity is of the order of 0.05 K. The radiances measured by CLIMAT-AV will be used to estimate the sea surface temperature.

### The HALO Aircraft and Its Instrumentation

The German high-altitude and long-range research aircraft HALO is a modified Gulfstream G550 business jet with a long endurance (more than 10 flight hours), a long range (about 8000 km), and a high ceiling (15.5 km) (Wendisch et al. 2016). In cooperation with the DLR and the Universities of Cologne, Hamburg, Leipzig and Munich, it will be equipped with an extensive set of remote sensing instrumentation (summarized in Table 2) including: the differential absorption and high-spectral-resolution lidar system (WALES, Water vApour Lidar Experiment in Space), HAMP (the HALO Microwave Package) which includes the cloud radar MIRA36 (36 GHz) and a microwave radiometer, the spectral imager specMACS, and an instrument system that measures spectrally resolved upward and downward solar radiances and irradiances (SMART). The payload also includes in situ measurements of the meteorological properties along the flight track (BAHAMAS), and the ability to launch dropsondes using the AVAPS system. To measure broadband upward and downward longwave radiances and remotely sensed surface temperatures, it is planned to add instruments such as the hemispheric broadband pyranometer (solar) and pyrgeometer (thermal infrared) and the CIMEL/CLIMAT-AV instruments used on the ATR-42. A cooled infrared imaging spectral camera will also be integrated.

MIRA36 is a commercially available METEK Ka-band (36 GHz) cloud research radar with polarization and Doppler capability to determine vertical velocity in clouds and precipitation. Together with microwave radiometers in the K, V, W, F, and G-band the MIRA36 is part of HAMP (Mech et al. 2014).

The lidar system WALES is a combined differential absorption and high-spectral-resolution lidar (HSRL) system developed and built at the Deutsches Zentrum für Luft- und Raumfahrt (Wirth et al. 2009). WALES is capable of nearly simultaneously emitting four wavelengths, three online and one offline, in the water vapour absorption band between 935 and 936 nm. The three online wavelengths achieve the necessary sensitivity needed for measurements over the whole range of tropospheric water vapour concentration. The vertical resolution of the raw data is 15 m. In addition to the 935-nm channel, the receiver is equipped with polarization-sensitive aerosol channels at 532 and 1064 nm, the first one with high-spectral-resolution capabilities using an iodine filter in the detection path (Esselborn et al. 2008). This allows for collocated measurements of humidity, optical depth, clouds and aerosol optical properties.

SpecMACS is an imaging cloud spectrometer developed at LMU (Ewald et al. 2016) consisting of two commercial spectral camera systems in the visible near-infrared (VNIR: 400–1000 nm) and in the shortwave infrared (SWIR: 1000–2500 nm). The nominal spectral resolution is 3 nm and 10 nm for the VNIR and for the SWIR, respectively. SpecMACS produces a spectrally resolved line image. For a flight level of about 10 km, a spatial resolution in the order of 10 m for cloud objects at a distance.

SMART (Spectral Modular Airborne Radiation Measurement System) consists of a set of spectral solar radiation sensors including radiances and irradiances (Wendisch et al. 2001; Ehrlich et al. 2008). All quantities are obtained for the wavelength range of 0.3–2.2 μm with spectral resolution of 2–16 nm full width of half maximum (FWHM), which is sufficient to analyse the spectral characteristics of spectral absorption bands of ice and liquid water. While the irradiance sensors provide spectral albedo at flight-level representative for a specific area, the measurement frequency of 2 Hz and the $2.1^{\circ }$ field of view of the radiance sensor allows identifying cloud inhomogeneities (about 200 m footprint for cloud top at 5 km and flight altitude of 10 km).

A similar payload was used during the NARVAL2 campaign and during the NAWDEX campaign of September–October 2016 over the North Atlantic.

## Appendix 2: Oceanographic Instrumentation

The thermal structure of the upper ocean has been historically observed from oceanographic ships and from expendable bathythermograph (XBT). Since the early 2000s, the advent of the Argo array of autonomous profiling floats has significantly increased the ocean sampling to achieve near-global coverage for the first time over the upper 1800 m and with a nominal resolution of 3 degrees in latitude/longitude. However, these new global observations are still very sparse and do not provide adequate measurements along boundaries of the oceans and within mesoscale eddies. This represents an acute weakness in our present understanding of ocean and atmosphere dynamics and their role in shaping the Earth’s climate variability and changes. To qualify and quantify the role of ocean eddies in the transport of water properties and in air–sea interactions, a number of oceanographic measurements will be necessary.

Ideally, in the year preceding the operational phase of EUREC^4^A we aim to deploy Argo profiling floats within anticyclonic eddies within the Guiana and North Equatorial currents. By associating these data with satellite observations of the atmosphere (clouds, water vapour, winds, etc), it will be possible to follow the joint evolution of eddies and lower atmospheric properties. Then, during the operational phase of EUREC^4^A, it will be appropriate to work with two ships measuring both air–sea fluxes, surface atmospheric properties and vertical profiles of temperature and salinity in the upper 2000 m of the ocean.

Ocean vertical profiles of temperature, salinity, currents, oxygen and other biogeochemical properties to also assess carbon related quantities will be acquired by a deep-reaching classical CTD rosette, equipped with sampling bottles and Acoustic Doppler Current profilers (ADCP; 150 or 300 kHz; one upward looking and one downward looking). To increase the sampling resolution, it will be very important to implement between CTD stations, at least for temperature, salinity and pressure, a very manageable and easy-to-use vertical profiler such as the Teledyne OceanScience Underway CTD/RapidCast (UCTD). On both ships, a microstructure vertical profiler would help infer turbulence linked with eddies and air–sea interactions.

The ships are equipped with an underway Thermosalinograph system (TSG) measuring near-surface temperature, salinity and, in many case, fluorescence. The vertical structure of ocean currents down to $> 1000 \hbox {m}$ depth along the ship track is recorded by ADCPs (38 and 75 kHz) mounted in the ships hull. Further instrumentations on the ships include standard marine atmospheric observing devices such as LIDARs (for vertical profiles of temperature, humidity and wind) and radiometers and precipitation gauges that allow to derive local ocean-atmosphere heat and freshwater fluxes. Operations of autonomous observing platforms such as underwater electric gliders and wave-gliders in regions of particular interest (e.g., frontal systems) will complement the observing efforts. The autonomous observing effort will start before the operational phase of EUREC^4^A and allow to observe the upper ocean conditions and their temporal and spatial evolution before, during and after the EUREC^4^A core field phase.
